# A cypovirus VP5 displays the RNA chaperone-like activity that destabilizes RNA helices and accelerates strand annealing

**DOI:** 10.1093/nar/gkt1256

**Published:** 2013-12-05

**Authors:** Jie Yang, Zhenyun Cheng, Songliu Zhang, Wei Xiong, Hongjie Xia, Yang Qiu, Zhaowei Wang, Feige Wu, Cheng-Feng Qin, Lei Yin, Yuanyang Hu, Xi Zhou

**Affiliations:** ^1^State Key Laboratory of Virology, College of Life Sciences, Wuhan University, Wuhan, Hubei 430072, China, ^2^State Key Laboratory of Pathogen and Biosecurity, Beijing Institute of Microbiology and Epidemiology, Beijing 100071, China and ^3^Department of Biochemistry, College of Life Sciences, Wuhan University, Wuhan, Hubei 430072, China

## Abstract

For double-stranded RNA (dsRNA) viruses in the family *Reoviridae*, their inner capsids function as the machinery for viral RNA (vRNA) replication. Unlike other multishelled reoviruses, cypovirus has a single-layered capsid, thereby representing a simplified model for studying vRNA replication of reoviruses. VP5 is one of the three major cypovirus capsid proteins and functions as a clamp protein to stabilize cypovirus capsid. Here, we expressed VP5 from type 5 *Helicoverpa armigera* cypovirus (HaCPV-5) in a eukaryotic system and determined that this VP5 possesses RNA chaperone-like activity, which destabilizes RNA helices and accelerates strand annealing independent of ATP. Our further characterization of VP5 revealed that its helix-destabilizing activity is RNA specific, lacks directionality and could be inhibited by divalent ions, such as Mg^2+^, Mn^2+^, Ca^2+^ or Zn^2+^, to varying degrees. Furthermore, we found that HaCPV-5 VP5 facilitates the replication initiation of an alternative polymerase (i.e. reverse transcriptase) through a panhandle-structured RNA template, which mimics the 5′-3′ cyclization of cypoviral positive-stranded RNA. Given that the replication of negative-stranded vRNA on the positive-stranded vRNA template necessitates the dissociation of the 5′-3′ panhandle, the RNA chaperone activity of VP5 may play a direct role in the initiation of reoviral dsRNA synthesis.

## INTRODUCTION

Reoviruses are a family of viruses (*Reoviridae*) that contain segmented double-stranded RNA (dsRNA) genome packaged by a single- or double-layered inner capsid and include several important pathogens that are responsible for diseases in humans, livestock animals, insects and plants (1). A common characteristic shared by reoviruses as well as many other dsRNA viruses is that their inner capsid contains several copies of RNA-dependent RNA polymerases (RdRPs) and mRNA-capping enzymes, and the process of reoviral positive-strand (+) RNA (mRNA) transcription from minus-strand (−) RNA, followed by mRNA capping, takes place within the reoviral inner capsid (1,2). The nascently synthesized and capped (+)RNA is then released into the cytoplasm of infected cells for protein translation. After that, reoviral (+)RNAs are encapsidated into newly assembled inner capsids, and are used by RdRPs as templates to synthesize genomic dsRNA segments within reoviral inner capsids (1,3,4). Thus, unlike most single-stranded RNA (ssRNA) viruses, reoviruses use their inner capsids as the machinery for viral RNA (vRNA) synthesis and the shield for evading host antiviral defenses (1,2,4).

Cypovirus (*cytoplasmic polyhedrosis virus*, CPV), which contains 10 genomic dsRNA segments, is one of the 15 genera within the family *Reoviridae* and is further classified into 16 types (cypovirus 1–16) based on the electrophoretic migration profiles of their genome segments (5) (http://www.ictvonline.org/virusTaxonomy.asp?version=2012). And the CPV particles are embedded in polyhedra for surviving unfavorable external environment (6). Unlike other multishelled reoviruses, cypovirus is the simplest genus of the family *Reoviridae* because it has only a single-layered capsid (7), and this characteristic makes it an ideal and simplified model for studying the mRNA/dsRNA synthesis and mRNA-capping mechanisms of reoviruses and probably other dsRNA viruses (8). For this reason, extensive molecular and structural studies have focused on CPVs in recent years (8–12). However, our knowledge about the molecular mechanisms that orchestrate the segmented dsRNA genome replication of CPVs and other reoviruses is still limited.

The CPV capsid comprises three major capsid proteins: VP1, VP3 and VP5 (12). Previous studies have shown that each CPV capsid contains 120 copies of VP1, 60 copies of VP3 and 120 copies of VP5. VP1 is the shell protein that forms the CPV capsid shell; VP3 is the mRNA-capping enzyme; and VP5 serves as the clamp protein to enhance the stability of the CPV capsid shell (10,12). In addition, one cypoviral RdRP, VP2, is located at the inner surface of the CPV capsid shell at each 5-fold axis (3,7). Moreover, Cheng and colleagues recently found that during cypoviral mRNA transcription, both VP1 and VP3 undergo conformational changes, resulting in an enlarged capsid chamber and a wider channel in the capsid for mRNA transport and capping (8). For VP5, no structural change has been detected (8), and it was not known whether VP5 contains any activity other than stabilizing capsid shell.

For RNA viruses including reoviruses, vRNA molecules require proper secondary and tertiary structures to form diverse *cis*-acting elements within their 5′-untranslated region, 3′-untranslated region or protein coding region, which are important for vRNA functions such as translation, replication and encapsidation (13–15). Moreover, the interactions (also termed ‘cyclization’) between the 5′- and 3′-termini of vRNAs have been recognized as an important prelude for efficient vRNA replication of many (+)RNA viruses like flaviviruses, (−)RNA viruses like hantaviruses and dsRNA viruses like reoviruses (16–18). For reoviruses, the 5′-3′ cyclization of reoviral (+)RNA forms a panhandle-like structure (19,20). This 5′-3′ panhandle is required for efficient dsRNA replication, probably by allowing reoviral RdRP to recognize the 3′-terminal of the (+)RNA template to initiate (−)RNA synthesis (18). On the other hand, like other 5′-3′ cyclized vRNAs (21), the panhandle should be disrupted when RNA replication proceeds on the cyclized template (18).

The formation and dissociation of RNA tertiary structures should be highly regulated for RNAs to function properly in various processes. However, correct folding or unfolding of RNAs is always challenging, since it is believed that RNAs would be easily trapped in local intermediate structures that are thermodynamically stable (15,22). In response, cells or viruses encode various RNA remodeling proteins, generally including adenosine triphosphate (ATP)-dependent RNA helicases and ATP-independent RNA chaperones, which are proposed to help overcome the thermodynamic barriers of kinetically trapped RNA molecules. RNA helicases are thought to be involved in most ATP-dependent structural rearrangements of RNAs (23). On the other hand, RNA chaperones are a heterogeneous group of proteins that are able to destabilize or unwind RNA helices and accelerate the formation of correctly folded RNA structures by helping misfolded RNAs escape kinetic traps. For the viruses having vRNA cyclization, both hantavirus nucleocapsid (N) protein and flavivirus core (capsid) protein are RNA chaperones, and hantavirus N has also been reported to unwind the 5′-3′ panhandle structure *in vitro* (21). However, no capsid protein of CPVs or other reoviruses has been found to be an RNA chaperone.

The type 5 *Helicoverpa armigera* cypovirus (HaCPV-5) was initially isolated by our laboratory in 2006 from a mixture of HaCPVs and can quickly cause lethal disease in *H. armigera*, which is one of the most serious agricultural pests in China (24). Thus far, the RNA genome of HaCPV-5 has been completely sequenced (24,25). HaCPV-5 RNA segment 8 contains a single open reading frame that encodes a 44-kDa protein, and further sequence and structural analysis using bioinformatic tools has revealed that this 44-kDa protein is the VP5 capsid protein (11,12,26). In this study, we expressed HaCPV-5 VP5 in a eukaryotic expression system and determined that this CPV VP5 possesses an RNA chaperone-like activity to ATP-independently destabilize RNA helices and accelerate strand annealing. Our further characterization of VP5 revealed that its helix-destabilizing activity is RNA specific, lacks directionality and could be inhibited by divalent metallic ions, such as Mg^2+^, Mn^2+^, Ca^2+^ or Zn^2+^, to various degrees. Moreover, we found that HaCPV-5 VP5 could facilitate the transcription initiation of an alternative polymerase (i.e. reverse transcriptase) through a CPV panhandle-structured RNA template, thereby strongly suggesting a direct role of the RNA chaperone activity of VP5 in the initiation of cypoviral dsRNA replication.

## MATERIALS AND METHODS

### Plasmid and recombinant baculovirus construction

Standard procedures were used for extraction of viral genome RNA and reverse transcription (RT)-polymerase chain reaction (25). A cDNA fragment of HaCPV-5 RNA segment 8 open reading frame (VP5) (Accession No. DQ178180) was inserted into the vector pFastBac™HTB-MBP that was originated from pFastBac™HTB (Invitrogen, Carlsbad, CA), in which the maltose binding protein (MBP) was N-terminally fused by us as previously described (27,28). Point mutations were introduced via polymerase chain reaction–mediated mutagenesis as described previously (29–31). The primers used in this study are shown in Supplementary Table S1. The constructed plasmids were subjected to the Bac-to-Bac system (Invitrogen) to express wild type and mutant MBP fusion VP5 (MBP-VP5) proteins.

### Expression and purification of recombinant fusion proteins

The expression and purification of recombinant MBP-VP5 and its derivatives as well as the negative-control protein MBP were carried out as previously described (28,32). Briefly, Sf9 cells were infected with the recombinant baculoviruses and harvested at 72 h postinfection. Cell pellets were resuspended, lysed by sonication and subject to centrifugation for 30 min at 11 000 *g* to remove debris. The protein in the supernatant was purified using amylose affinity chromatography (New England BioLabs, Ipswich, MA), according to the manufacturer’s protocol. Fractions containing the recombinant protein were combined, followed by concentration using an Amicon Ultra-15 membrane column (Millipore, Schwalbach, Germany). After that, the buffer was exchanged to 25 mM 2-[4-(2-hydroxyethyl)-1-piperazinyl] ethanesulfonic acid (HEPES)-KOH (pH 7.5), 50 mM NaCl, and the protein was stored at −70°C in aliquots. All proteins were quantified by the Bradford method.

### Sodium dodecyl sulphate-polyacrylamide gel electrophoresis and western blot analysis

Sodium dodecyl sulphate-polyacrylamide gel electrophoresis (SDS-PAGE) and western blot assays were performed as described previously (32). The anti-MBP polyclonal antibody was purchased from New England BioLabs, and used at the dilutions of 1:10 000.

### Modeling analysis of HaCPV-5 VP5

The 3D structure of HaCPV-5 VP5 was modeled by submitting its amino acid sequence to the HMMSTR/Rosetta server [from Robetta, University of Washington (http://robetta.bakerlab.org/)] (26). Five models were obtained, and the best one was chosen as a template based on its score, assessed by submitting it to the SWISS-MODEL Server [from Swiss Institute of Bioinformatics and the Biozentrum, University of Basel, Switzerland (http://swissmodel.expasy.org)]. The figure of the modeled HaCPV-5 VP5 3D structure was drawn by PyMOL program 1.1 (DeLano Scientific LLC, South San Francisco, CA) from coordinate file. The surface representation of modeled VP5 on CPV particle are drawn by PyMOL 1.1 based on the atomic cryoEM structure of BmCPV capsid (PDB number 3IZX) by replacing BmCPV VP5 with HaCPV-5 VP5.

### Preparation of oligonucleotide helix substrate

RNA or DNA helix substrates were prepared by annealing two complementary nucleic acid strands. One strand was labeled at 5′-end with hexachloro fluorescein (HEX) (Takara, Dalian), and the other strand was unlabeled. The two strands were mixed in a proper ratio, and annealed through heating and gradually cooling. All unlabeled DNA strands were synthesized by Invitrogen, and HEX-labeled DNA and RNA strands were purchased from Takara. Unlabeled RNA strands were synthesized by us from the *in vitro* transcription using T7 RNA polymerase (Promega, Madison, WI). The transcribed RNA strands were purified by Poly-Gel RNA Extraction Kit (Omega bio-tek, Guangzhou, China) according to the manufacturer’s instruction.

Standard RNA helix substrate was annealed with RNA1 and RNA3, D*/R substrate was annealed with DNA1 and RNA3, R*/D substrate was annealed with RNA1 and DNA2, D*/D substrate was annealed with DNA1and DNA2, 3′-tailed substrate was annealed with RNA1 and RNA4, 5′-tailed substrate was annealed with RNA1 and RNA5, and blunt-ended substrate was annealed with RNA1 and RNA2. The 3′-tailed RNA helix substrates with different lengths of 3′-tails were prepared by annealing RNA1 with and RNA4, RNA6, RNA7, RNA8 and RNA9. The sequences of all DNA and RNA strands are listed in Supplementary Table S2.

### Gel mobility shift assay

Gel mobility shift was performed in 25 mM HEPES-KOH (pH 7.5), 100 mM NaCl, in a volume of 10 μl with indicated amount of protein and 0.1 pmol of ssRNA (RNA1) or blunt-ended dsRNA (RNA1/RNA2). RNA1 is HEX-labeled. Reactions were incubated for 30 min at room temperature. For the competition experiments, unlabeled ssRNA or dsRNA competitor was added together with the HEX-labeled RNA probe (RNA1) to the binding reaction. The reactions were terminated by the addition of 2.5 μl 5× sample buffer [20 mM Tris–HCl (pH 7.5), 30% glycerol and 0.1% bromophenol blue]. The nucleic acid–protein complexes were separated by electrophoresis on 2% agarose gels, and gels were scanned by a Typhoon 9200 imager (GE Healthcare, Piscataway, NJ). The values of Hill coefficient, an indicator of the cooperativity of RNA binding, were calculated by applying Hill transformation to the RNA binding data obtained in repeated experiments.

### Cross-linking assays

Chemical cross-linking assays were performed as previously described (29). MBP-tagged proteins were chemically cross-linked in the cross-linking buffer (100 mM HEPES [pH 7.4], 50 mM NaCl and final 0.01% [vol/vol] glutaraldehyde) for 30 min. The complexes were then analyzed via 10% SDS-PAGE and western blots. To detect which form of MBP-VP5 binds RNA, MBP-VP5 were first cross-linked with the HEX-labeled RNA1 probe using a 254-nm ultraviolet (UV) light for 10 min, and the light source was 5 cm away from the samples. After that, the samples were subjected to chemical cross-linking as described above. The complexes were then analyzed via 10% SDS-PAGE and scanned by a Typhoon 9200 imager (GE Healthcare).

### Nucleic acid helix destabilizing assay

The standard helix destabilizing assay was performed as previously described (28,32) with minor modifications. In brief, 10 pmol of protein and 0.1 pmol of helix substrate were added to a mixture containing a final concentration of 25 mM HEPES-KOH (pH 8.0), 0.01% bovine serum albumin (BSA), 50 mM NaCl, 1 mM MgCl_2_, 5 mM Dithiothreitol (DTT) and 5 U RNasin (Promega) and incubated at 37°C for 60 min unless otherwise indicated. The reactions were terminated by adding 5 U proteinase K and 2.5 μl 5× loading buffer [100 mM Tris–HCl (pH7.5), 50% glycerol and bromophenol blue]. Mixtures were electrophoresed on 12% native-PAGE gels. Gels were scanned with a Typhoon 9200 imager (GE Healthcare). The ratio of released single strands versus the total substrates was quantified with ImageQuant software.

### RNA strand hybridization assay

The standard RNA strand hybridization assay was performed as previously described (32). In brief, indicated amount of MBP-VP5 was incubated with HEX-labeled and unlabeled RNA strands (0.1 pmol each strand) in 37°C in a buffer containing 50 mM HEPES-KOH (pH 8.0), 2.5 mM MgCl_2_, 2 mM DTT, 0.01% BSA and 5 U RNasin. The reaction was terminated and analyzed as described above. For the hybridization assay of the stem-loop structured RNA strands, the sequences of the two stem-loop RNA strands were indicated in Figure 9A, and the stem-loop structures were predicted by mfold (http://mfold.rna.albany.edu/?q=mfold). For the hybridization of the HEX-labeled 46-nt and unlabeled 146-nt RNA strands, their sequences are listed in Supplementary Table S2. Then mixtures were also resolved on 12% native-PAGE gels (Figure 9) or 8% native-PAGE gels (Figure 10), following by scanning with a Typhoon 9200 imager (GE Healthcare).

**Figure 1.**
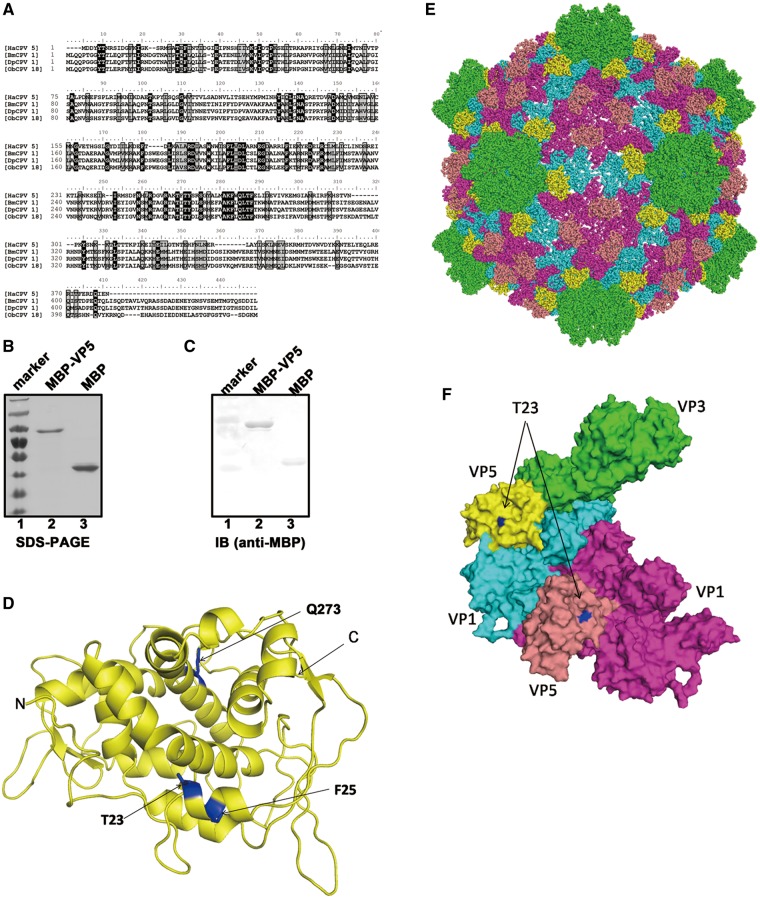
(**A**) The amino acid sequence alignment of VP5 proteins of HaCPV-5, BmCPV-1, *Dendrolimus punctatus* CPV-1 (DpCPV-1) and *Operophtera brumata* CPV-18 (ObCPV-18). Multiple sequence alignments were generated using ClustalX. (**B**) Electrophoresis analysis of purified MBP-VP5 and MBP alone. Proteins were loaded onto a 12% SDS-PAGE gel and then visualized via Coomassie blue staining. Lane 1, protein molecular mass markers; lane 2, purified MBP-VP5; lane 3, purified MBP. (**C**) The purified MBP-VP5 and MBP alone were subjected to SDS-PAGE, followed by western blot analysis with anti-MBP polyclonal antibodies. (**D**) The 3D structure of HaCPV-5 VP5 was modeled by the HMMSTR/Rosetta server, and drawn by PyMOL as described in ‘Materials and Methods’ section. The three mutation sites as well as N- and C-termini were labeled as indicated. (**E** and **F**) The surface representations of modeled VP5, which indicate the orientation of VP5 on the CPV particle (E), or in the asymmetric unit formed by VP1, VP3 and VP5 (F). The T23 on the VP5 is shown in blue. Color-coded by protein subunits, VP3 is in yellow, VP1 has two conformers in cyan and magenta, while VP5 in yellow and salmon. The maps of CPV particle and the asymmetric unit are drawn by PyMOL based on the atomic cryoEM structure of BmCPV capsid (PDB number 3IZX) by replacing BmCPV VP5 with HaCPV-5 VP5.

### RT assay

The RT assay was previously described by Mir *et al.* (21) with some modifications. Briefly, the structured RNA mimicking cypovirus panhandle was generated using 36 nt from the 5′-end and 30 nt from the 3′-end of HaCPV-5 RNA segment 8. The sequences of the panhandle and DNA primer were indicated in Figure 12A. The mixture of 0.1 pmol panhandle, 5 pmol DNA primer and 0.5 mM deoxynucleoside triphosohates (dNTPs) with digoxigenin (DIG)-dUTP was incubated with indicated amount of MBP-VP5 or MBP alone without heating and cooling, while for the positive control, the mixture was heated at 68°C for 3 min and placed on ice to allow the annealing of the primer to the panhandle. After incubation, 4 μl of the RT buffer, 1 μl of 0.1 M DTT, 5 U RNasin and 0.5 μl M-MLV reverse transcriptase (Promega) were supplemented and reacted at 25°C for 30 min. Reactions were terminated by heating at 80°C for 5 min. The samples were analyzed on 6% urea page and northern blot. The northern blot was performed as previously described (29).

## RESULTS

### HaCPV-5 VP5 is an RNA binding protein

Among HaCPV-5 RNA segments, segment 8 encodes a 44-kDa protein that is homologous with VP5 proteins of other CPVs based on amino acid sequences (Figure 1A) but shares no sequence homology with any other non-VP5 CPV proteins (data not shown). To further compare this protein with other CPV VP5 proteins, we modeled its 3D structure by submitting its amino acid sequence to the HMMSTR/Rosetta server (26) and found that its modeled structure is highly similar to that of BmCPV-1 VP5 (Figure 1D), which has been solved and extensively studied (33). Altogether, these results show that this protein is the VP5 capsid protein of HaCPV-5.

**Figure 2.**
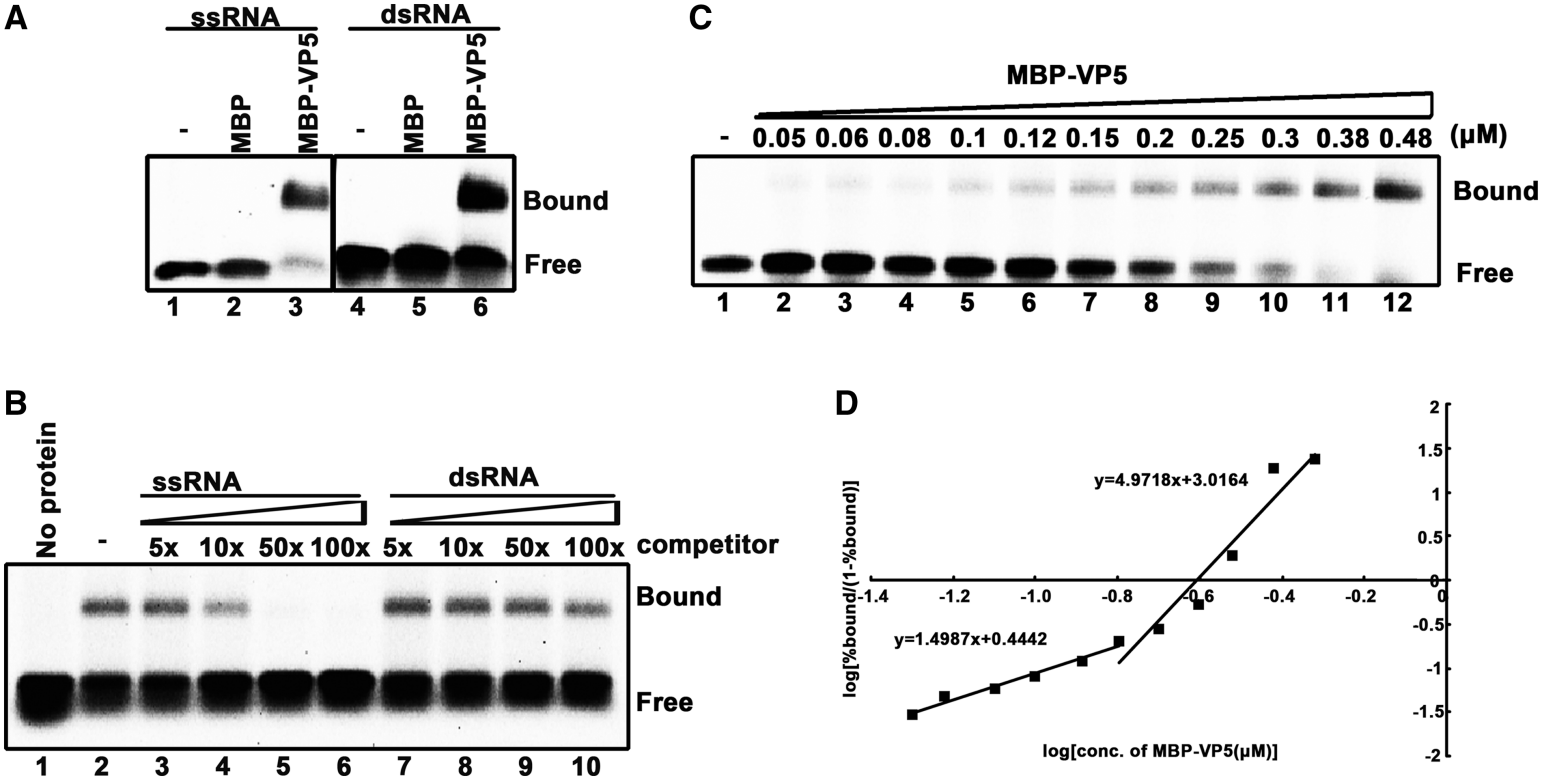
VP5 binds to RNA in a cooperative manner. (**A**) A gel mobility shift assay was performed to evaluate the dsRNA or ssRNA binding capacity of MBP-VP5. Ten picomoles MBP-VP5 was incubated with 0.1 pmol HEX-labeled ssRNA (RNA1) or dsRNA (RNA1/RNA2) substrate for 30 min. Lanes 1 and 4, no protein supplemented; lanes 2 and 5, 10 pmol MBP supplemented; lanes 3 and 6, 10 pmol MBP-VP5 supplemented. Protein-bound and free RNA strands are indicated. (**B**) 0.1 pmol HEX-labeled ssRNA probe and 2 pmol MBP-VP5 were incubated with unlabeled competitor ssRNA or dsRNA at the indicated increasing concentrations (in 5-, 10-, 50-, 100-folded excess over the amount of HEX-labeled ssRNA). The bound complexes were analyzed in a gel mobility shift assay. (**C**) Gel mobility shift assays were performed by incubating the indicated increasing concentrations (0.05–0.45 μM) of MBP-VP5 with 0.1 pmol RNA1 probe. (**D**) The RNA binding data in (C) were quantified, and a Hill transformation was applied. The Hill coefficients of the RNA binding of VP5 at low and high protein concentrations are indicated.

To determine the potential function of HaCPV-5 VP5, we expressed this protein as an N-terminal maltose binding fusion protein (MBP-VP5) in a eukaryotic (baculovirus) expression system and then purified the protein (Figure 1B and C). Because many capsid proteins of RNA viruses have RNA binding activity (21,34,35), we sought to determine whether VP5 could also bind to RNA. To this end, a gel mobility shift assay with a HEX-labeled 24-nt ssRNA (RNA1) was conducted. MBP-VP5 did bind to RNA1 (Figure 2A, lane 3), showing that VP5 has ssRNA binding activity. Moreover, to determine whether VP5 also has dsRNA binding activity, we constructed the RNA helix by annealing HEX-labeled RNA1 with a nonlabeled RNA (RNA2) that is complementary to RNA1 and found that VP5 efficiently shifted the RNA helix (Figure 2A, lane 6). After determining that MBP-VP5 can bind to both ssRNA and dsRNA, it is intriguing for us to examine whether this protein has a higher affinity for ssRNA or for dsRNA. To this end, RNA probe competition assays were performed. Briefly, 0.1 pmol HEX-labeled ssRNA probe and 2 pmol MBP-VP5 were incubated in the presence of increasing amounts of unlabeled ssRNA (RNA10) or dsRNA (RNA10/RNA2). As shown in Figure 2B, only the ssRNA competitor efficiently competed with HEX-labeled RNA1, while dsRNA had a minimal competing effect, indicating that MBP-VP5 has a higher affinity for ssRNA.

Moreover, we incubated 0.01 μM of HEX-labeled RNA1 with increasing concentrations of MBP-VP5 (0.05–0.48 μM), and our result showed that MBP-VP5 binds to ssRNA in a dose-response manner (Figure 2C). The RNA binding data were then quantified, and a Hill transformation was applied to determine whether the binding of MBP-VP5 to ssRNA is cooperative [a >1 Hill coefficient indicates positive cooperativity (36,37)]. As shown in Figure 2D, MBP-VP5 at low concentrations (0.05–0.16 μM) had a Hill coefficient value of ∼1.499, whereas high protein concentrations (0.2–0.48 μM) had a higher Hill coefficient value of ∼4.972. These results indicate that VP5 binds to ssRNA in a cooperative manner. Taken together, these results show that HaCPV-5 VP5 has both ssRNA and dsRNA binding capacity, has higher affinity for ssRNA and binds ssRNA in a cooperative manner. Besides, we also examined if MBP-VP5 can bind to DNA, and our results showed that this protein also has ssDNA and dsDNA binding activities (Supplementary Figure S1).

**Figure 3.**
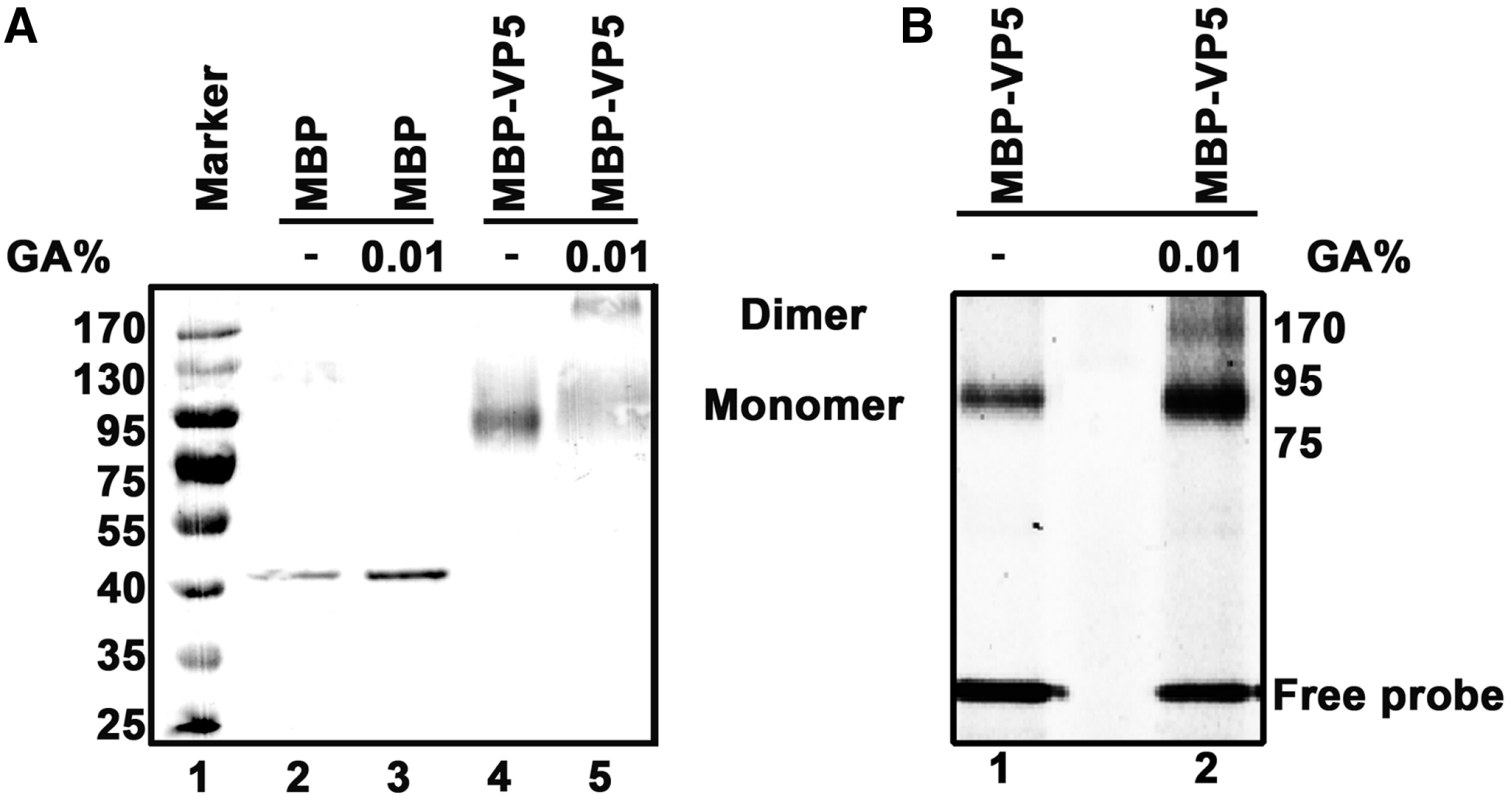
Monomeric and dimeric MBP-VP5 bind to ssRNA. (**A**) MBP alone (lane 3) or MBP-VP5 (lane 5) was incubated in a chemical cross-linking buffer containing 0.01% glutaraldehyde (GA) as indicated, and then analyzed via 10% SDS-PAGE, followed by western blots with anti-MBP antibodies as described in ‘Materials and Methods’ section. Lanes 2 and 4, MBP alone and MBP-VP5 in the absence of 0.01% GA. (**B**) MBP-VP5 was firstly UV cross-linked with HEX-labeled RNA1 probe, and then subjected to chemical cross-linking by incubating with 0.01% GA. The samples were analyzed via 10% SDS-PAGE, followed by scanning using a Typhoon 9200 imager to visualize HEX-labeled RNA probe. The dimeric and monomeric forms of MBP-VP5 are indicated.

After determining the RNA binding activity of VP5, we ought to examine the form of MBP-VP5 in solution. To this end, the *in vitro* chemical cross-linking assay was performed, followed by SDS-PAGE and western blot analysis. Our result showed that MBP-VP5 has both monomeric and dimeric forms in solution (Figure 3A). Moreover, this result was further confirmed by the gel filtration assay (Supplementary Figure S2). Then, it is intriguing to determine which form of MBP-VP5 binds to RNA. To this end, MBP-VP5 was incubated with HEX-labeled RNA1 probe and then subjected to UV cross-linking. The purpose of this step is to label the VP5 proteins that are binding to RNA. After that, the samples were subjected to chemical cross-linking with 0.01% [vol/vol] glutaraldehyde, and analyzed via SDS-PAGE and scanned by a Typhoon 9200 to visualize the presence of HEX-labeled RNA. Our results showed that both monomeric and dimeric forms of MBP-VP5 can bind to ssRNA *in vitro* (Figure 3B), indicating that the RNA binding activity of VP5 is independent of its monomeric or dimeric form.

### The VP5 protein can destabilize the RNA helix

**Figure 4.**
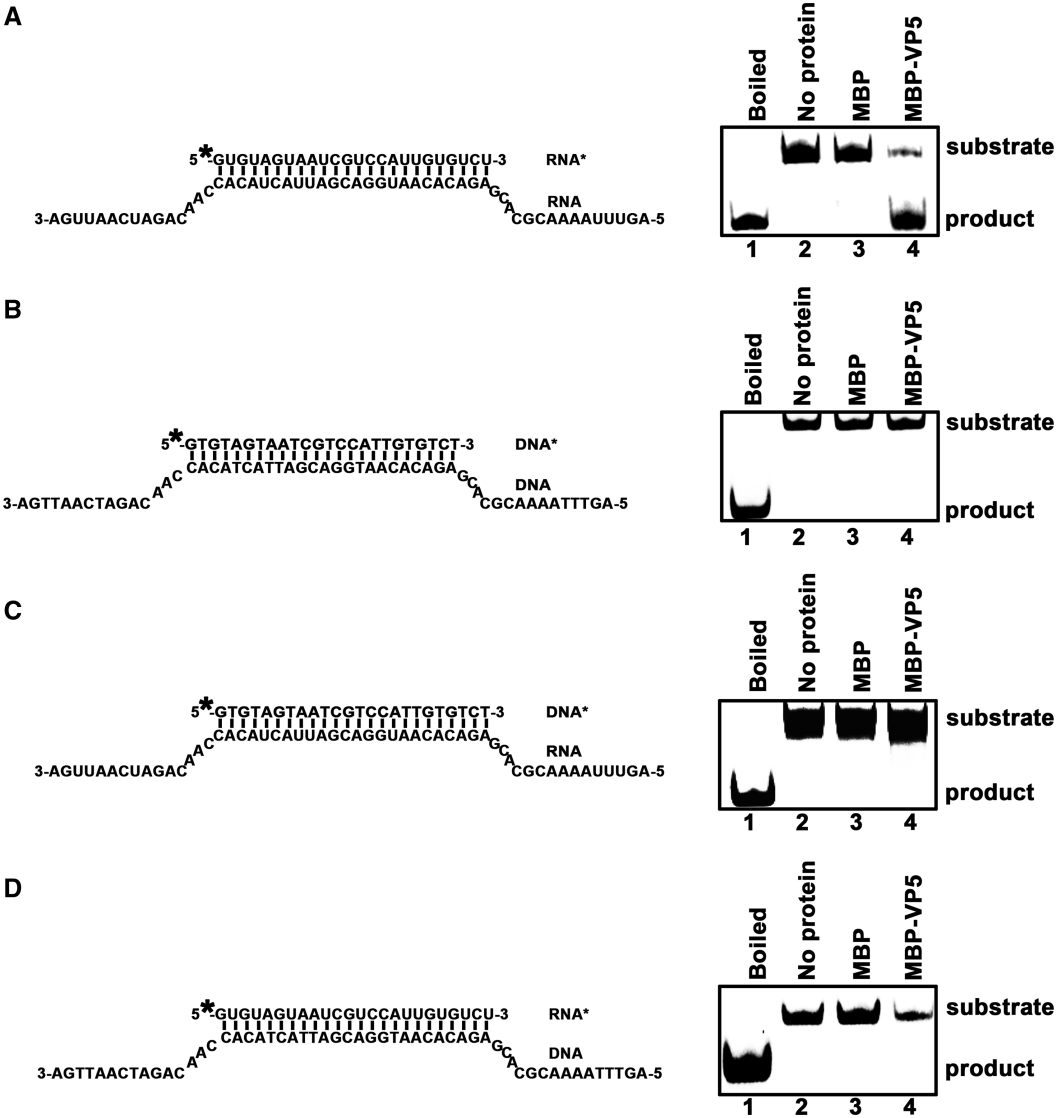
VP5 destabilizes RNA helices. Purified MBP-VP5 was incubated with (**A**) standard RNA helix (R*/R substrate), (**B**) DNA helix (D*/D substrate), (**C**) DNA/RNA hybrid helix (D*/R substrate) or (**D**) RNA/DNA hybrid helix (R*/D substrate) as illustrated in the left panels. Asterisk indicates HEX-labeled strands. The preparations of these destabilizing substrates are indicated in ‘Materials and Methods’ section. Helix substrate (0.1 pmol) was incubated in standard reaction mixtures in the presence or absence of 10 pmol MBP-VP5 as indicated, and the destabilizing activity was determined via gel electrophoresis and scanning on a Typhoon 9200. Lane 1, boiled reaction mixture without protein supplementation; lane 2, reaction mixture without protein supplementation; lane 3, reaction mixture with MBP alone; and lane 4, reaction mixture with MBP-VP5.

The finding that HaCPV-5 VP5 contains both ssRNA and dsRNA binding activities led us to question whether this protein also has nucleic acid helix-destabilizing activity like flavivirus core (capsid) and hantavirus N do (21,35). For this purpose, the HEX-labeled RNA1 and a long nonlabeled 54-nt RNA (RNA3) were annealed to generate a standard RNA helix substrate with both 5′ (15 bases) and 3′ (15 bases) single-stranded tails (Figure 4A). The helix-destabilizing assay was performed by incubating the standard RNA helix substrate with purified MBP-VP5 in the standard destabilizing reaction mixture and then evaluating the substrate via gel electrophoresis. The HEX-labeled RNA strand was released from the RNA helix substrate in the presence of MBP-VP5 (Figure 4A, lane 4), whereas the RNA helix was stable when MBP alone was added to the reaction mixture (lane 3). The boiled helix substrates were used as the positive controls for helix destabilization in this (lane 1) and the subsequent assays. These results indicate that VP5 has RNA helix-destabilizing activity.

Because some viral proteins with RNA helix-destabilizing activities can also unwind DNA helices or RNA–DNA hybrids, we sought to determine whether VP5 can destabilize nucleic acid helices containing DNA. To this end, we constructed three different helix substrates: D*/D (Figure 4B) by annealing a short HEX-labeled 24-nt DNA (DNA1) and a long nonlabeled 54-nt DNA (DNA2); D*/R (Figure 4C) by annealing HEX-labeled DNA1 and the long nonlabeled RNA3; and R*/D (Figure 4D) by annealing HEX-labeled RNA1 and nonlabeled DNA2. Each substrate was incubated with MBP-VP5 under the conditions described in Figure 4A. MBP-VP5 was unable to destabilize any of these DNA-containing helices (Figure 4B–D), showing that the helix-destabilizing activity of VP5 is RNA specific. Interestingly, we previously found that VP5 can bind both RNA and DNA (Figure 2A and Supplementary Figure S1), suggesting that the protein binding to nucleic acids is the prerequisite but not the only determinant for helix destabilization.

**Figure 5.**
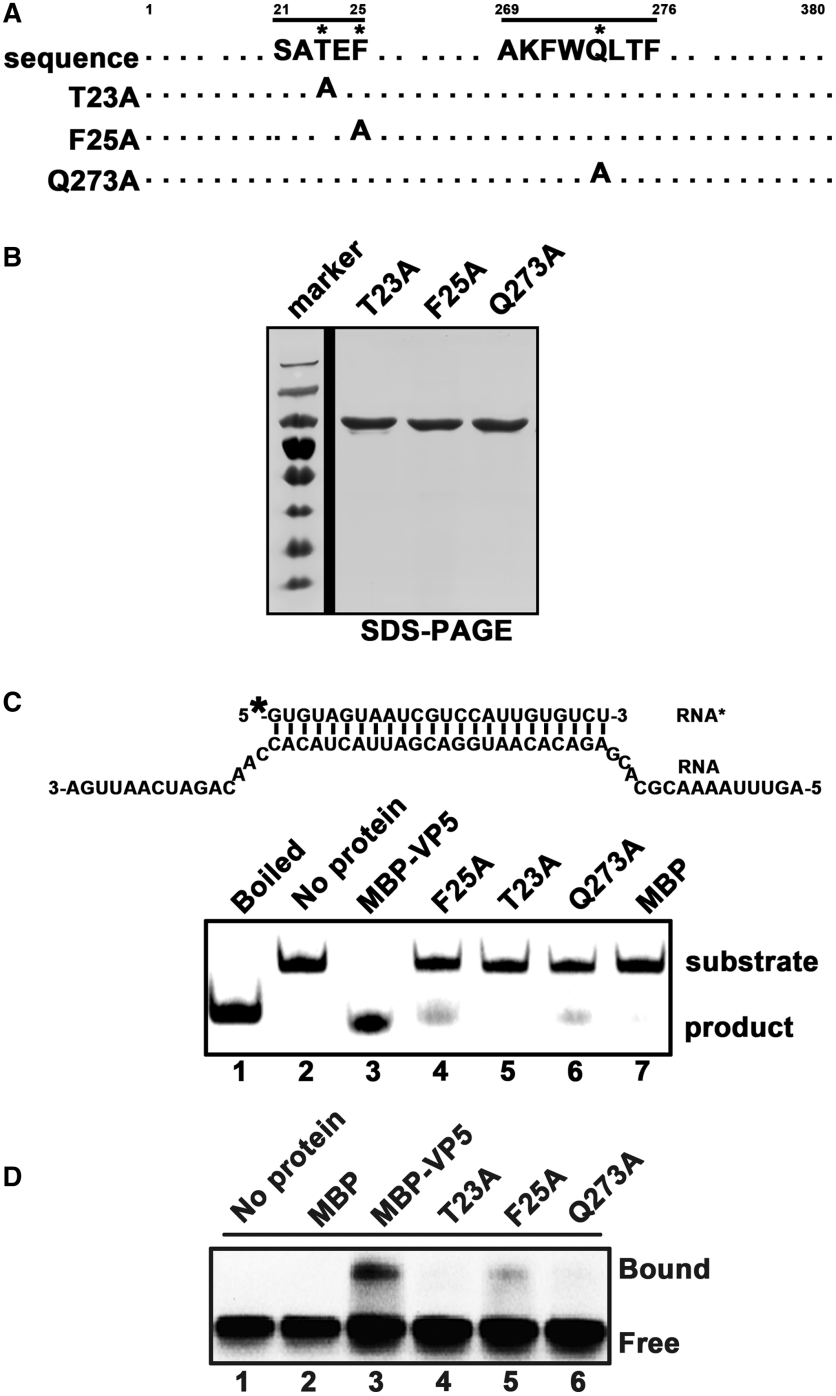
Mutational analysis of the RNA helix-destabilizing activity of VP5. (**A**) Illustration of the mutagenesis strategy. Asterisk indicates sites of replacement with alanine. (**B**) Expressed and purified MBP-VP5 mutants were subjected to 12% SDS-PAGE followed by Coomassie brilliant blue R250 staining. (**C**) Helix substrate (0.1 pmol; RNA1/RNA3; upper panel) was reacted with 10 pmol MBP-VP5 wild-type and mutants as indicated (lanes 3–6). Boiled reaction mixture (lane 1) was used as positive control, and the reaction mixture with no protein (lane 2) or supplemented with MBP alone (lane 7) was used as a negative control. (**D**) Gel mobility shift assays were performed by incubating 0.1 pmol HEX-labeled RNA1 with 10 pmol MBP-VP5 wild type and mutants as indicated (lanes 3–6). Reaction mixture without protein supplementation (lane 1) or with MBP alone (lane 2) was used as a negative control.

To further confirm the helix-destabilizing activity of VP5, we generated point mutations (T23A, F25A and Q273A) at the highly conserved residues within the consensus regions of CPV VP5 (Figure 5A). These mutants were expressed in a eukaryotic expression system as MBP-fusion proteins, purified (Figure 5B) and then assessed for their helix-destabilizing activities. Our results showed that the F25A or Q273A mutation dramatically reduced the destabilization of the standard RNA helix substrate (Figure 5C, lanes 4 and 6), whereas the T23A mutation almost abolished the unwinding activity of VP5 (Figure 5C, lane 5). Moreover, we examined the RNA binding activities of these mutants, and found that these mutations resulted in the loss or dramatic decrease of RNA binding (Figure 5D). Based on the modeled surface representation of these mutant residues on VP5 (Figure 1D–F), T23 is located on the surface of VP5, while F25 and Q273 residues are located within the protein, suggesting that the strategies of these mutations to inhibit RNA binding and helix destabilizing activities of VP5 are different.

### Characterization of the RNA helix-destabilizing activity of VP5

**Figure 6.**
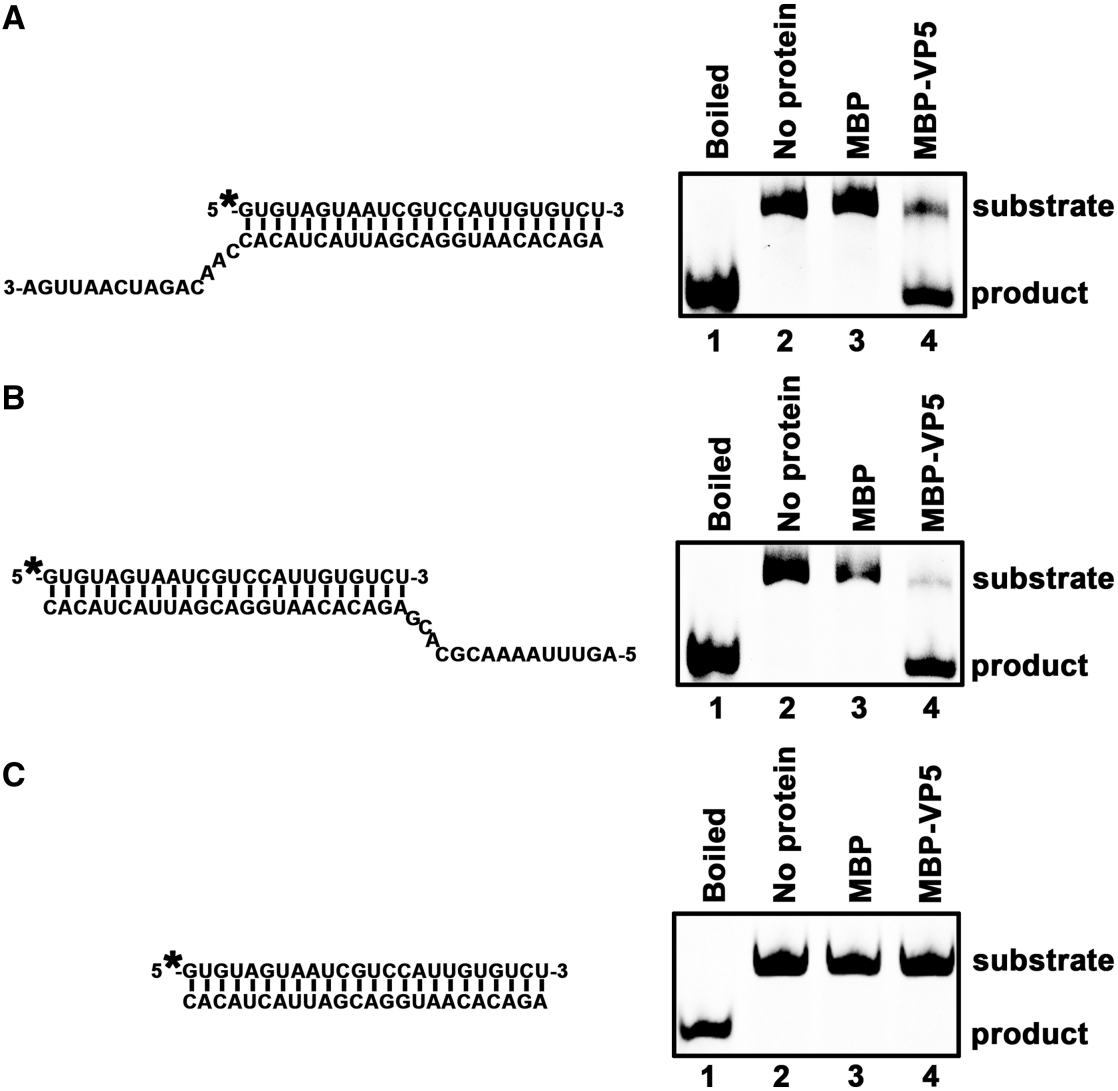
VP5 destabilizes RNA helices in a bidirectional manner. Purified MBP-VP5 was incubated with (**A**) 3′-tailed RNA helix (RNA1/RNA4), (**B**) 5′-tailed RNA helix (RNA1/RNA5) or (**C**) blunt-ended RNA helix (RNA1/RNA2) as illustrated in the left panels. Asterisk indicates the HEX-labeled strand (RNA1). The preparations of these destabilizing substrates are indicated in ‘Materials and Methods’ section. RNA helix substrate (0.1 pmol) was incubated in standard reaction mixtures in the presence or absence of 10 pmol MBP-VP5 as indicated, and the destabilizing activity was determined via gel electrophoresis and scanning on a Typhoon 9200. Lane 1, boiled reaction mixture without protein supplementation; lane 2, reaction mixture without protein supplementation; lane 3, reaction mixture with MBP alone; and lane 4, reaction mixture with MBP-VP5.

To characterize the helix-destabilizing activity of VP5, we designed three different RNA helix substrates: one containing a 3′ single-stranded tail (15 bases), one containing 5′ single-stranded tail (15 bases) and one with blunt ends (Figure 6A–C, left panels). Each substrate was incubated with MBP-VP5 in the standard destabilizing reaction mixture. Our results showed that both the 3′-tailed and 5′-tailed RNA helices could be efficiently unwound by MBP-VP5 (Figure 6A and B), whereas the blunt-ended RNA helix could not be unwound (Figure 6C). These experiments were independently repeated several times by us. Overall, these results show that HaCPV-5 VP5 destabilizes RNA helices in both 3′-to-5′ and 5′-to-3′ directions.

**Figure 7.**
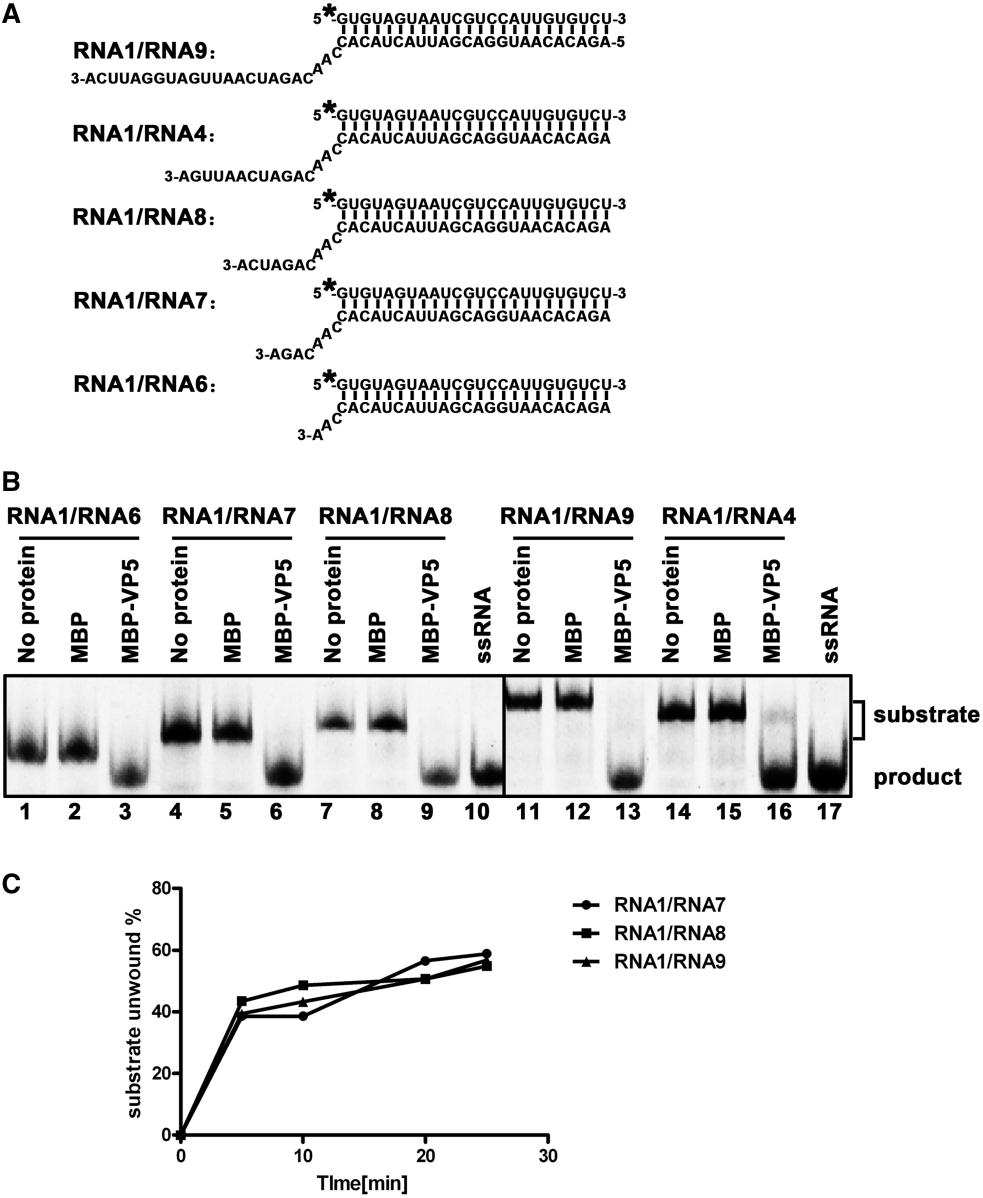
The length of 3′-tail of RNA helices has no obvious impact on the helix-destabilizing activity of VP5. (**A**) Schematic illustration of RNA helix substrates with the indicated lengths of 3′-tails. The shorter strand (RNA1) was HEX labeled. (**B**) Indicated 3′-tailed RNA helix substrate (0.1 pmol) was incubated with 10 pmol MBP-VP5, and the destabilizing activity was determined via gel electrophoresis and scanning on a Typhoon 9200. Reaction mixture without protein supplementation (lanes 1, 4, 7, 11 and 14) or with MBP alone (lanes 2, 5, 8, 12 and 15) was used as a negative control, and ssRNA was loaded to indicate the position of free ssRNA strand in the gel (lanes 10 and 17). (**C**) Three different RNA helix substrates (RNA1/RNA7, RNA1/RNA8 and RNA1/RNA9) were incubated with 10 pmol MBP-VP5 for 0, 5, 10, 20 and 25 min. The unwinding activity was quantified and plotted as the percentage of the released RNA from the total RNA helix substrate (Y-axis) at each time point (X-axis).

Next, we sought to determine the impact of the length of the single-stranded tail on its helix-destabilizing activity. To this end, a series of RNA helix substrates with different lengths (3–23 nt) of 3′ single-stranded tails were generated (Figure 7A), and unwinding assays were performed by incubating 10 pmol MBP-VP5 with 0.1 pmol indicated helix substrates for 60 min (Figure 7B) or for 5 different time points (0, 5, 10, 20 and 25 min) (Figure 7C). Our results showed that all these single-stranded 3′-tails could similarly support the helix destabilization by VP5 and the length of 3′-tail had no much effect on the helix destabilization. This phenomenon is consistent with our previous observation of *Ectropis obliqua* picorna-like virus (EoV) nonstructural protein 2C, which also displays RNA chaperone activity (32).

**Figure 8.**
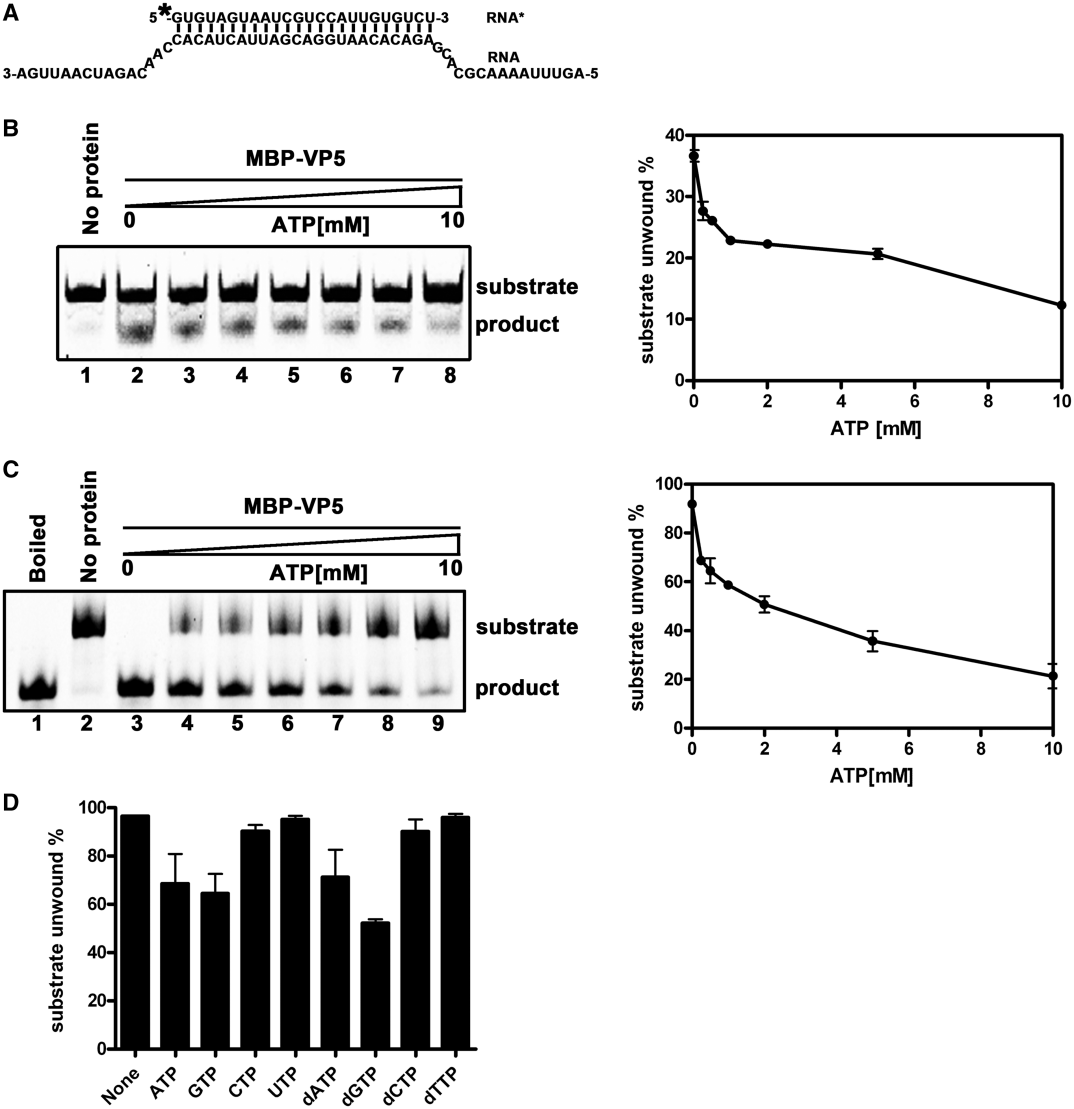
Increasing concentrations of ATP inhibited the helix-destabilizing activity of VP5. (**A**) The standard RNA helix substrate (RNA1/RNA3) is indicated, and asterisk indicates the HEX-labeled strand. (**B**) The helix substrate (0.1 pmol) was incubated with 10 pmol MBP-VP5 for 10 min in the standard reaction condition in the absence (lane 2) or presence of increasing concentrations of ATP (lanes 3–8; 0.25, 0.5, 1.0, 2.0, 5.0 and 10.0 mM ATP). (**C**) The helix substrate (0.1 pmol) was incubated with 10 pmol MBP-VP5 for 60 min in the standard reaction condition in the absence (lane 3) or presence of increasing concentrations of ATP (lanes 4–9; 0.25, 0.5, 1.0, 2.0, 5.0 and 10.0 mM ATP). The destabilizing activity was determined via gel electrophoresis and scanning on a Typhoon 9200. The unwinding activity was plotted as the percentage of the released RNA from the total RNA helix substrate (Y-axis) at each ATP concentration (X-axis) (B and C, right panels). Error bars represent standard deviation (SD) values from three separate experiments. (**D**) The helix substrate (0.1 pmol) was incubated with 10 pmol MBP-VP5 for 60 min in the standard reaction condition in the presence of different NTP or dNTP at 0.5 mM as indicated. The unwinding activity was graphed as the percentage of the released RNA from the total substrate (Y-axis) in the presence of different NTP or dNTP (X-axis). Error bars represent SD values from three separate experiments.

Previously, we established the RNA helix-destabilizing activity of HaCPV-5 VP5 in the absence of ATP. Thus, we sought to determine whether the presence of ATP has any effect on the unwinding activity of VP5. For this purpose, the standard RNA helix substrate with both 3′ and 5′ tails (Figure 8A) was incubated with MBP-VP5 in the presence of increasing concentrations (0.25–10 mM) of ATP for 10 min (Figure 8B) or 60 min (Figure 8C). In either condition, the presence of ATP had no positive effect on the unwinding activity of VP5 (Figure 8B and C); in contrast, higher ATP concentrations (>0.25 mM) actually inhibited RNA helix destabilization in a dose-response manner (Figure 8B, lanes 3–8; 8C, lanes 4–9). These experiments were independently repeated several times, and the gradual inhibitory effect of increasing concentrations of ATP was plotted (Figure 8B and C, right panels).

After determining that ATP has an inhibitory effect on helix destabilization by VP5, we sought to determine whether other nucleoside triphosphates (NTPs) or dNTPs have similar or different effects. To this end, the destabilizing activity of VP5 was assessed in the presence of ATP, GTP, CTP, UTP, dATP, dGTP, dCTP or dTTP at a final concentration of 0.5 mM. As shown in Figure 8D, ATP, dATP, GTP or dGTP had an obvious inhibitory effect on the destabilizing activity of VP5, but other NTPs or dNTPs had negligible inhibitory effect. Altogether, these data further confirm the ATP independence of VP5 helix-destabilizing activity, thereby excluding the possibility that VP5 may contain ATP-dependent RNA helicase activity.

### VP5 destabilizes structured RNA strands and stimulates the annealing of RNA strands

**Figure 9.**
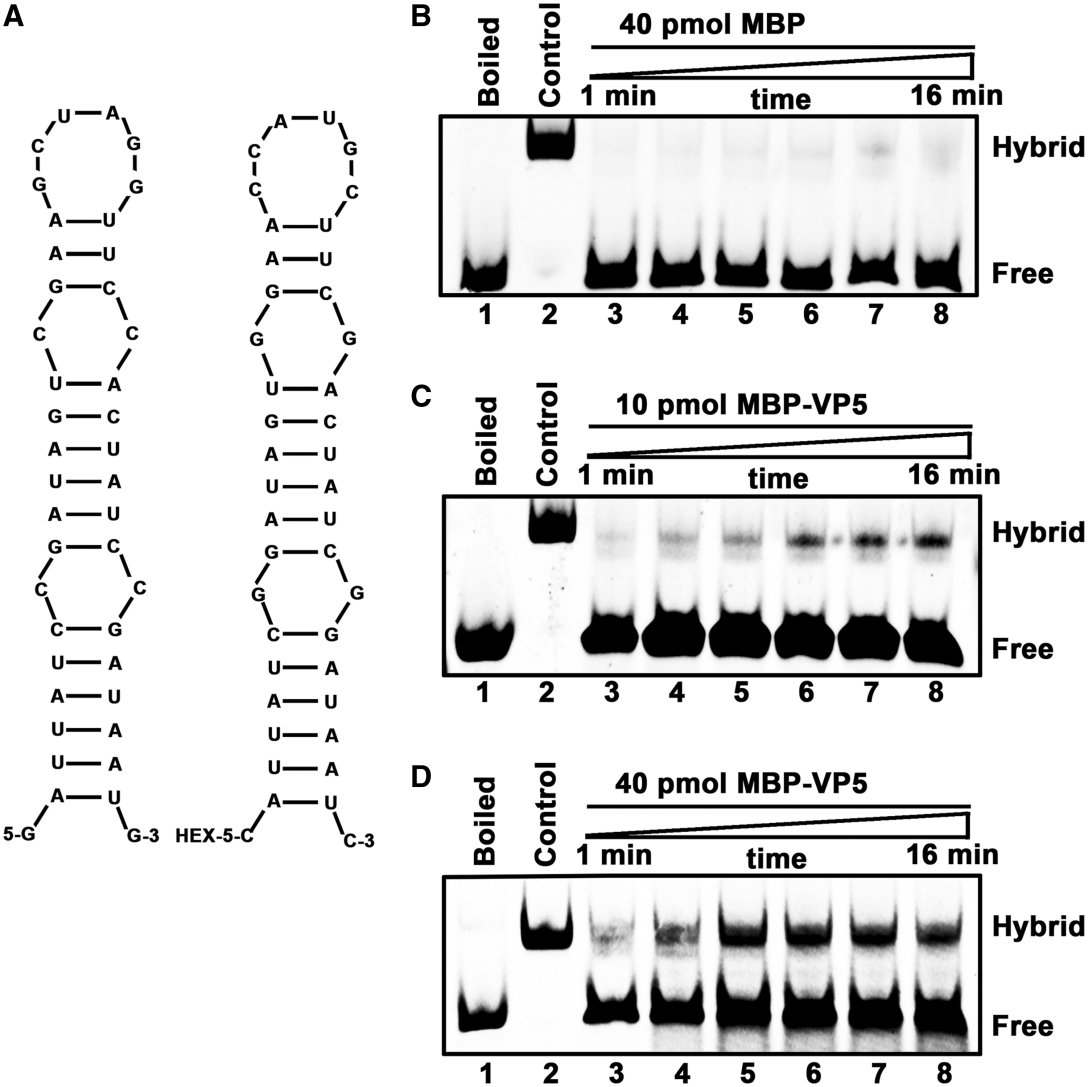
VP5 destabilizes stem-loop structured RNA strands. (**A**) Schematic illustrations of the stem-loop structures of the two complementary 42-nt RNA substrates. HEX labeling is indicated (right). (**B–D**) The two strands were mixed (0.1 pmol each strand) and reacted in the presence of (B) 40 pmol MBP, (C) 10 pmol MBP-VP5 or (D) 40 pmol MBP-VP5 for the indicated time (lanes 3–8; 1, 2, 4, 8, 10 and 16 min). For (B–D), the mix of the two strands was boiled (lane 1) or preannealed (lane 2) as a negative or positive control. The hybridized and free strands are indicated.

To characterize the activity of VP5 in destabilizing RNA secondary structures, we adapted a canonical assay, which was initially developed by DeStefano and colleagues for measuring the helix-destabilizing and annealing acceleration activities of vRNA chaperones (38–40), for this cypoviral VP5. For this purpose, two 42-nt complementary RNA strands that form defined stem-loop structures were used (Figure 9A), and one strand was 5′ HEX labeled (Figure 9A, right). HEX-labeled (0.1 pmol) and nonlabeled (0.1 pmol) strands were mixed and incubated with MBP alone or MBP-VP5 in the standard destabilizing mixture, and then the hybridization of the two complementary strands was measured via gel electrophoresis (Figure 9B–D). In the presence of MBP alone, little spontaneous hybridization was detected according to increased reaction time (Figure 9B, lanes 3–8), whereas the presence of 10 pmol VP5 promoted the hybridization of the two RNA strands (Figure 9C, lanes 3–8). Moreover, when we increased the amounts of MBP-VP5 to 40 pmol, a more dramatic stimulation of the RNA strand annealing was observed (Figure 9D, lanes 3–8). These data show that VP5 contains the helix-destabilizing activity that can unwind RNA secondary structures and stimulate the formation of more stable RNA hybrids.

**Figure 10.**
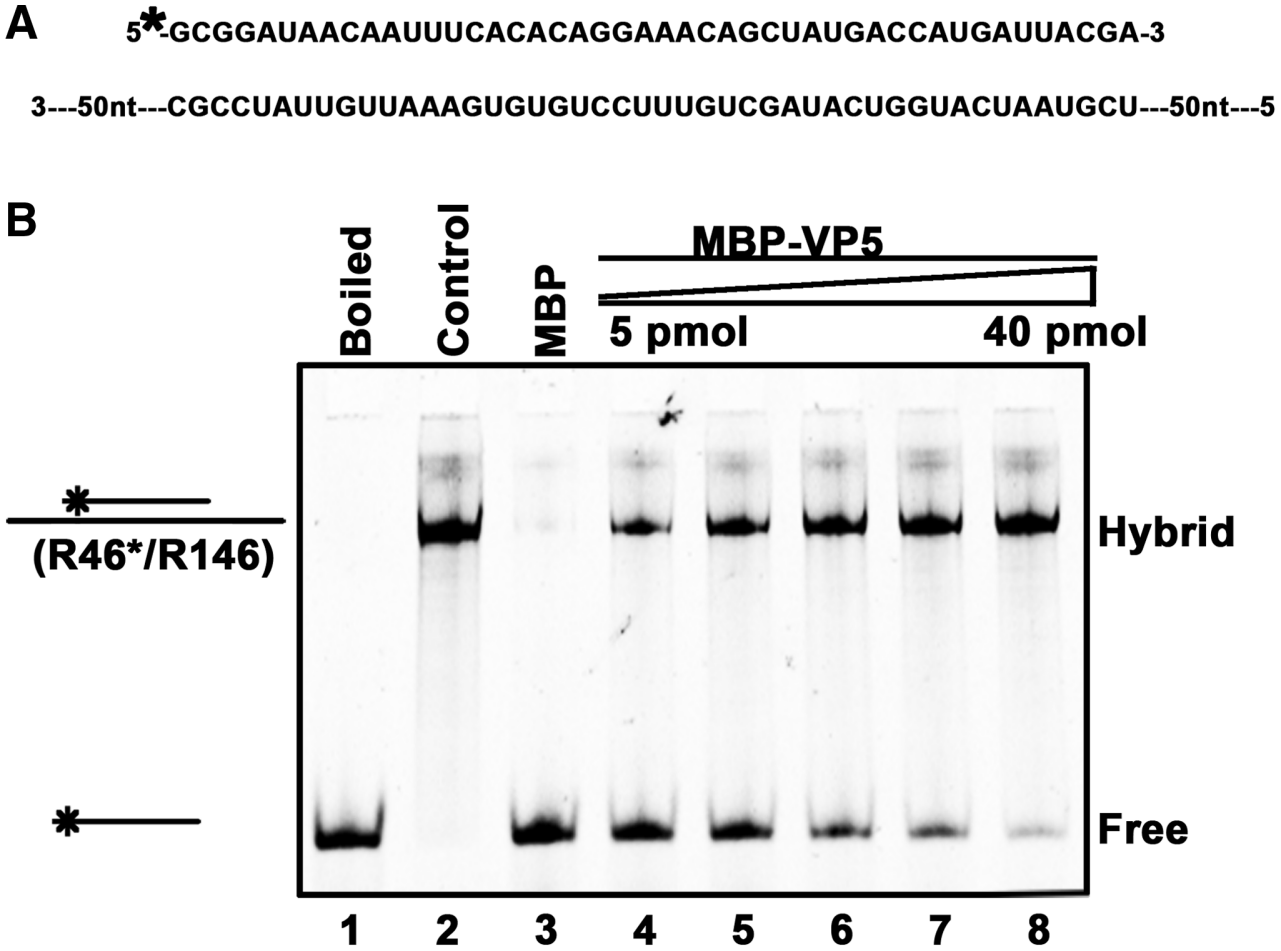
VP5 stimulates RNA strand annealing. (**A**) Schematic illustrations of the HEX-labeled 46-nt RNA strand and the nonlabeled longer complementary 146-nt RNA strand. Asterisk indicates the HEX-labeled strand. (**B**) Equal amounts (0.1 pmol each) of the two strands were mixed and reacted with increasing amounts of MBP-VP5 for 15 min. Lane 1, the mixture was boiled before being loaded onto the gel as a negative control; lane 2, the two strands were preannealed using a thermal cycler as a positive control; lane 3, the reaction with MBP alone as a negative control; and lanes 4–8, the reactions with 5, 10, 15, 20 and 40 pmol MBP-VP5.

To further determine the strand-annealing stimulation activity of VP5, we generated a shorter HEX-labeled RNA strand (46 nt in length) and a longer nonlabeled complementary RNA strand (146 nt in length; Figure 10A). Equal amounts (0.1 pmol) of these two strands were incubated for 15 min in the presence of MBP alone or MBP-VP5. Gel electrophoresis was conducted to detect the annealing of the two complementary RNA strands. The annealing of the RNA strands in the presence of MBP alone was almost undetectable (Figure 10B, lane 3), whereas the presence of 5 pmol MBP-VP5 dramatically stimulated strand annealing (Figure 10B, lane 4). Moreover, nucleic acid chaperone normally functions well when its amount is in large excess over that of its substrate (21). To determine whether this notion also applies to VP5, we incubated increasing amounts (5–40 pmol) of MBP-VP5 with the two RNA strands (0.1 pmol each). As shown in Figure 10B (lanes 4–8), increasing the amount of MBP-VP5 led to stronger stimulating effects on strand annealing in a dose-response manner. Taken together, our data show that HaCPV-5 VP5 contains RNA chaperone-like activity that can destabilize RNA helices and stimulate RNA strand annealing.

### Characterization of the optimal biochemical reaction conditions for the RNA helix-destabilizing activity of VP5

**Figure 11.**
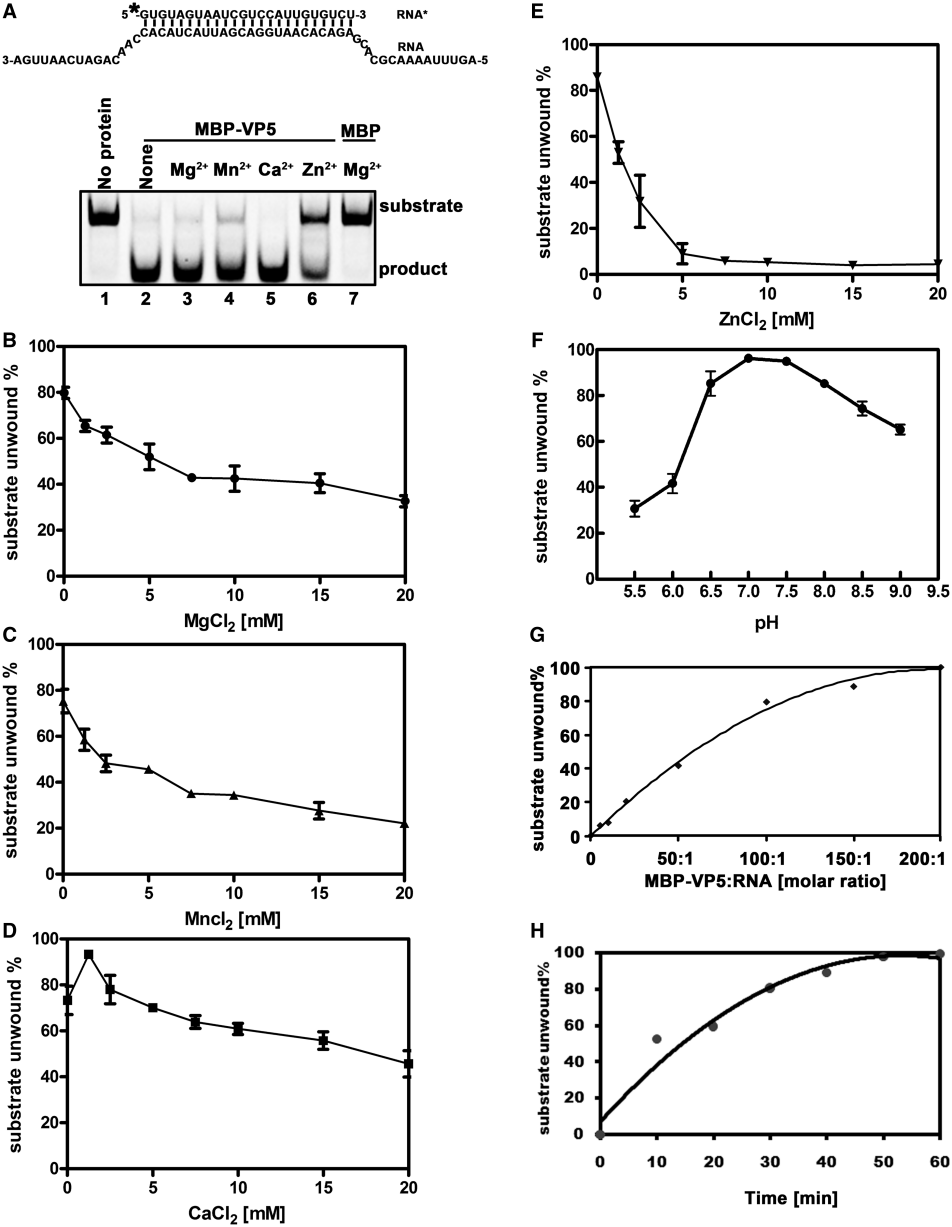
Optimal biochemical reaction conditions for the RNA helix-destabilizing activity of VP5. (**A**) Standard RNA helix substrate (RNA1/RNA3; 0.1 pmol) as illustrated in upper panel was incubated with 10 pmol MBP-VP5 in the absence (lane 2) or presence of 2.5 mM indicated divalent metal ions (lanes 3–6) for 60 min. Reaction mixture without protein addition (lane 1) or with MBP alone (lane 7) was used as a negative control. Asterisk indicates the HEX-labeled strand. (**B–E**) Standard RNA helix substrate (RNA1/RNA3; 0.1 pmol) was incubated with 10 pmol MBP-VP5 in the presence of increasing concentrations of (B) MgCl_2_, (C) MnCl_2_, (D) CaCl_2_ or (E) ZnCl_2_ as indicated for 30 min. (**F**) Standard RNA helix substrate (0.1 pmol) was incubated with 10 pmol MBP-VP5 at indicated pH values in the absence of divalent ions for 30 min. (**G**) Standard RNA helix substrate (0.1 pmol) was incubated with MBP-VP5 at indicated VP5/RNA molar ratios for 30 min. (**H**) 10 pmol MBP-VP5 was incubated with 0.1 pmol standard RNA helix substrate (at the molar ratio of 100:1) for indicated time intervals. For (B–H), the unwinding activity was plotted as the percentage of the released RNA from the total RNA helix substrate as Y-axis at indicated ion concentrations (B–E), pH values (F), VP5/RNA molar ratios (G) or incubation time intervals (H) as X-axis. Error bars represent SD values from three separate experiments.

To further characterize the biochemical properties of VP5, we evaluated its RNA helix-destabilizing activity under various conditions with different divalent metallic ions, ion concentrations and pH values. First, we assessed the requirement of divalent metallic ions, including Mg^2+^, Mn^2+^, Ca^2+^ and Zn^2+^, on the helix-destabilizing activity of VP5. In this experiment, 0.1 pmol standard RNA duplex substrate with both 5′ and 3′ tails (Figure 11A, upper panel) was incubated with 10 pmol MBP-VP5 in the presence of 2.5 mM Mg^2+^ Mn^2+^, Ca^2+^ or Zn^2+^ for 60 min. The presence of 2.5 mM Mg^2+^ or Ca^2+^ did not effect helix destabilization (Figure 11A [lower panel], lane 2 versus lane 3 or 5); on the other hand, the presence of 2.5 mM Mn^2+^ slightly inhibited the helix-destabilizing activity of VP5 (lane 4), and the inhibitory effect of Zn^2+^ was more dramatic (lane 6).

Next, we assessed the impact of Mg^2+^, Mn^2+^, Ca^2+^ or Zn^2+^ concentrations on the helix-destabilizing activity of MBP-VP5. Of note, because the RNA duplex substrate was completely unwound by VP5 in the presence of Mg^2+^ or Ca^2+^ when incubated for 60 min, a shorter incubation time (30 min) was used in the subsequent experiments to measure the effects of varying ion concentrations. Our results showed that the destabilizing activity was optimal at 0 mM for Mg^2+^ or Mn^2+^ (Figure 11B and C), and the increase of Mg^2+^ or Mn^2+^ concentrations from 0 to 20 mM led to a gradual decrease in the helix-destabilizing activity of MBP-VP5. Interestingly, this observation also excludes the possibility that the inhibitory effect of higher ATP concentrations (>0.25 mM) on VP5-mediated helix destabilization (Figure 8) was due to the chelation of Mg^2+^, which could be caused by adding large quantities of ATP or other NTPs (32,41), since lower Mg^2+^ concentrations actually enhance helix destabilization.

For Ca^2+^, the helix-destabilizing activity of MBP-VP5 was optimal at 1.25 mM, and the increase of Ca^2+^ concentrations from 1.25 to 20 mM also led to a gradual decrease in the RNA helix destabilization (Figure 11D). Our results showed that the inhibitory effect of Zn^2+^ on helix destabilization was more dramatic than that of the other three divalent ions, and the helix-destabilizing activity of VP5 was almost abolished when the concentration of Zn^2+^ reached 5 mM (Figure 11E). Moreover, all these divalent metallic ions conferred inhibitory effects in a dose-response manner.

Furthermore, we sought to determine the optimal pH value for the helix-destabilizing activity of VP5. VP5 prefers a neutral or mildly basic pH, as the reaction conditions were optimal at pH 7.0–7.5 (Figure 11F).

Last, we assessed the effects of the molar ratio of VP5 versus RNA duplex substrate as well as the incubation time on the destabilizing activity of VP5. At a molar ratio of 100:1, the helix-unwinding efficiency of VP5 reached ∼80%, and VP5 completely unwound the RNA duplex substrates at a ratio of 200:1 (for 30 min incubation; Figure 11G). A subsequent experiment showed that the helix-destabilizing activity of VP5 (at the molar ratio of 100:1) was dependent on the incubation time (Figure 11H).

### VP5 facilitates RT initiation through the CPV panhandle structure *in vitro*

**Figure 12.**
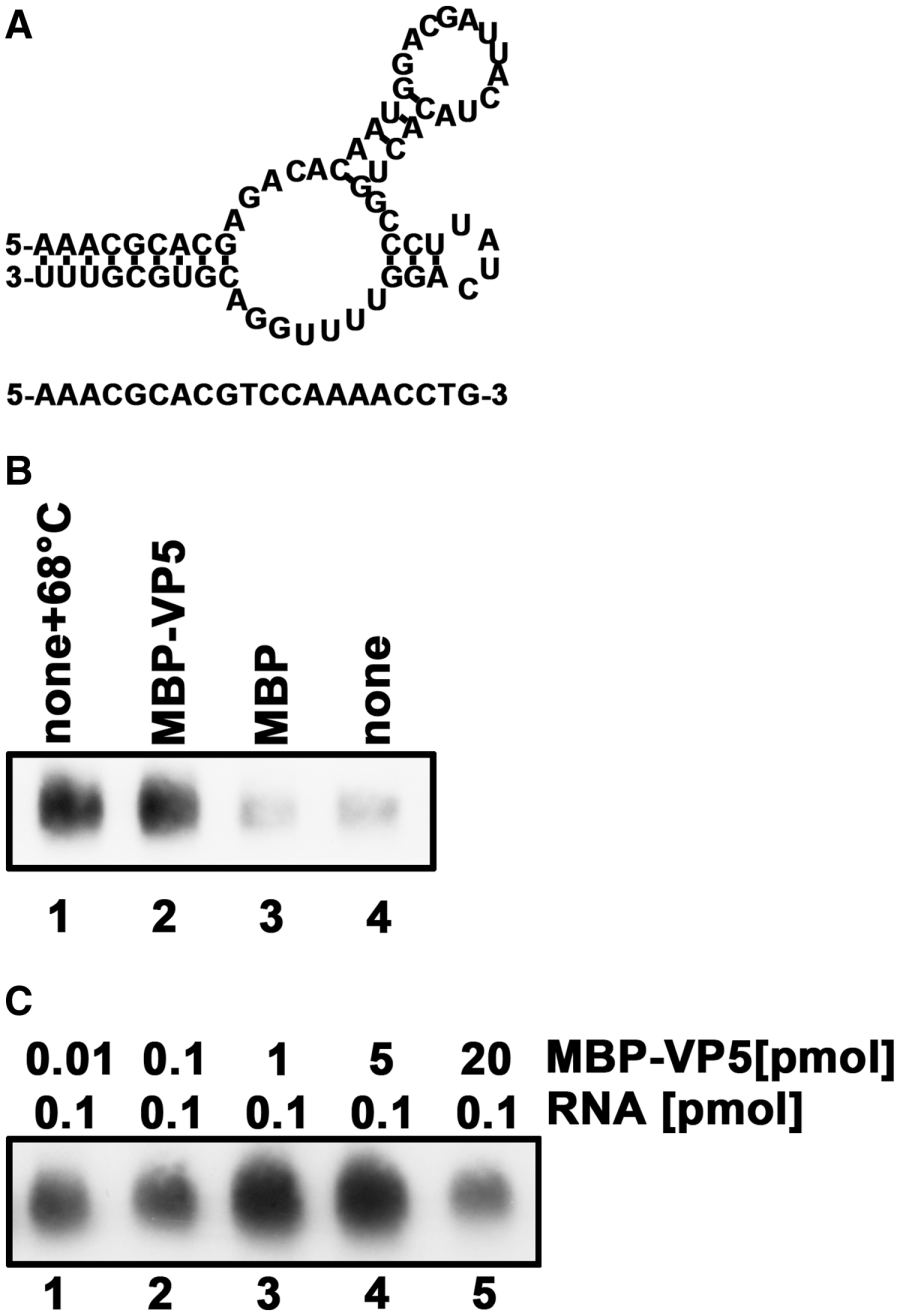
VP5 facilitates RT initiation via a CPV panhandle. (**A**) Schematic illustrations of the predicted panhandle structure formed by 36 bases from the 5′-end and 30 bases from the 3′-end of HaCPV-5 RNA segment 8 (upper panel) and the primer complementary to the 3′-end of the panhandle (lower panel). (**B**) The RT reactions were conducted using the RNA panhandle structure and the DNA primer in the presence of M-MLV reverse transcriptase for 30 min at 25°C in the absence (lanes 1 and 4) or presence of MBP-VP5 (lane 2) or MBP alone (lane 3). For lane 1, the panhandle and primer were prehybridized via a thermal annealing treatment at 68°C before the reaction. (**C**) The RT reactions were conducted using the indicated amounts of RNA panhandles in the presence of indicated amounts of MBP-VP5 for 30 min at 25°C. The samples were analyzed on 6% urea PAGE, followed by northern blotting.

After determining the RNA chaperone activity of VP5, we sought to determine its potential role in CPV RNA replication. Since the destabilization of the 5′-3′ panhandle structure of CPV (+)RNA should occur before or when the (−)RNA synthesis proceeds on the cyclized (+)RNA template (18), it is plausible that VP5 functions to unwind the reoviral 5′-3′ panhandle to allow replication initiation at the accessible 3′-end by an RdRP. To assess this possibility, we adapted a canonical assay, which was developed by Panganiban and colleagues for assessing the ability of hantavirus N protein to facilitate RNA replication initiation through the hantaviral panhandle structure (21), for VP5. We used this assay to determine whether VP5 can enhance *in vitro* primer-dependent replication initiation by an alternative polymerase (i.e. reverse transcriptase) through the cypoviral panhandle structure. To this end, a structured RNA mimicking the cypoviral panhandle structure was constructed using 36 nt from the 5′-end and 30 nt from the 3′-end of HaCPV-5 RNA segment 8, and then used as the template for RT (Figure 12A, upper panel). Moreover, because RT reactions normally involve thermal cycles at 68°C to denature and anneal the RNA template with a primer, the RT reactions were carried out at 25°C in the presence or absence of MBP-VP5 to assess the potential role of the VP5’s RNA chaperone activity to unwind panhandle structure of the RNA template. Here, DIG-labeled dUTP was supplemented into the reaction mixture for visualizing the RT products. As shown in Figure 12B, the presence of VP5 dramatically stimulated the synthesis of RT products (lane 2), as did the thermal annealing treatment at 68°C (lane 1); on the other hand, the reaction product was barely detectable in the absence of VP5 (Figure 12B, lanes 3 and 4).

To further confirm the capacity of VP5 to facilitate RT through the panhandle structure, we conducted the reactions at varying molar ratios of VP5 to the panhandle-structured template. Our data showed that the stimulating effect of VP5 on the RTs was optimal at molar ratios of 10:1–50:1 (Figure 12C, lanes 3 and 4).

Taken together, these results strongly suggest that VP5 has a direct role in CPV dsRNA synthesis by destabilizing the 5′–3′ panhandle structure to promote the accessibility of the 3′-end of the (+)RNA template for replication initiation.

## DISCUSSION

As one of the three major CPV capsid proteins, VP5 is also named as clamp protein or large protrusion protein. Previous studies have demonstrated that 120 copies of VP5 are located on the surface of the CPV capsid, exist in two conformations and function as molecular clamps to interact with and tie together neighboring VP1 shell proteins, thus enhancing the stability of the CPV capsid (8,33). Moreover, the structural homologs of CPV VP5 are commonly present in the inner capsids of other turreted reoviruses, including orthoreovirus σ2 and aquareovirus VP6, which also function as molecular clamps to stabilize inner capsids (12,33,42). However, whether VP5, a major component of CPV dsRNA replication machinery, and other reoviral clamp proteins contain activities other than structural stabilization was not known. In this report, we show that the VP5 protein from HaCPV-5 is a novel RNA chaperone that destabilizes RNA helices and stimulates strand annealing in an ATP-independent manner, implying a direct role of VP5 in the replication of CPV dsRNA.

It is believed that the vRNA molecules of many RNA viruses could be kinetically trapped in incorrect/inactive intermediate structures, thereby probably requiring RNA chaperones to facilitate proper RNA folding and efficient vRNA replication/translation (14,43,44). So far, HIV-1 nucleocapsid (NC), Vif and Tat, flavivirus core, hantavirus N, poliovirus 3AB, coronavirus nucleocapsid (N), tombusvirus p33, hepatitis D virus small delta antigen and EoV 2C have been determined to contain RNA chaperone activities (14,32). The list of virus-encoded RNA chaperones is still growing, and our current study adds HaCPV-5 VP5 as a new member of it.

In the family *Reoviridae*, CPV VP5 may not be the only RNA chaperone, as rotavirus nonstructural protein 2 (NSP2), which is a multifunctional enzyme involved in rotaviral dsRNA replication, was previously shown to contain ATP-independent nucleic acid helix-destabilizing activity (45). As observed with CPV VP5, the helix-destabilizing activity of rotavirus NSP2 has no nucleotide sequence specificity and lacks unwinding directionality. It is generally believed that the helix-destabilizing activity of RNA chaperones normally requires a large excess of protein over RNA helix substrates (32,39), and this notion also applies to both CPV VP5 and rotavirus NSP2. The optimal unwinding activity of VP5 was observed at the protein-to-nucleic acid molar ratio of ∼100:1–200:1, whereas the optimal activity of rotavirus NSP2 was detected at much higher molar ratios of ∼1000:1–2000:1 (45).

On the other hand, some features of rotavirus NSP2 and CPV VP5 are different. For instance, VP5 binds to both RNA and DNA and exhibits RNA-specific destabilizing activity (Figure 4), whereas NSP2 is an ssRNA binding protein and can unwind both RNA–RNA and DNA–RNA helix substrates (45,46). The difference between VP5 and NSP2 implies that these two proteins may not use the same mechanism to destabilize RNA helices. Moreover, because no previous study has determined whether rotavirus NSP2 possesses strand annealing acceleration activity, an important characteristic of RNA chaperones, further characterization of NSP2 is needed to determine whether this protein can be recognized as an RNA or nucleic acid chaperone.

Both CPV VP5 and rotavirus NSP2 are components of reoviral RNA replication machinery: the former is a major CPV capsid protein, and the latter is associated with the rotaviral RdRP (12,47). There is no homology between rotavirus NSP2 and any CPV structural or nonstructural proteins; on the other hand, CPV VP5 has no sequence similarity with any rotavirus proteins. Moreover, as a ‘nonturreted’ reovirus, rotavirus does not encode clamp proteins (48). These observations led us to question whether CPV or rotavirus contains extra RNA chaperone or helix-destabilizing protein, except VP5 or NSP2, respectively. However, since it is not uncommon for a virus (e.g. HIV-1) to encode multiple RNA chaperones, the possibility that a reovirus contains more than one RNA chaperones cannot be ruled out, and it would be interesting for us or others to investigate this issue in the future.

During the RNA replication of reoviruses, (+)-vRNAs (mRNAs) are encapsidated into the nascently assembled inner capsids and are then used by reoviral RdRPs as replication templates to synthesize genomic dsRNA segments within the inner capsids (1,2,4). As seen with (−)-vRNA of hantavirus, reoviral (+)-vRNA can be cyclized, and its 5′- and 3′-ends undergo base pairing to form a panhandle-like structure (49). Previous studies revealed that the panhandle structure of cyclized reoviral (+)-vRNA is required for efficient dsRNA synthesis probably by recruiting RdRPs to the 3′-end of the (+)-vRNA template (18). Moreover, like other 5′-3′ cyclized vRNAs, before or when RNA replication initiates, the vRNA panhandle structure should be dissociated to make the 3′-end of the template accessible by RdRPs (18). Considering that hantavirus N, a nucleocapsid protein and RNA chaperone, can efficiently unwind the 5′-3′ panhandle of cyclized hantaviral (−)-vRNA and subsequently promote transcription/replication initiation (21,50), we propose that the RNA chaperone activity of CPV VP5 is directly involved in the initiation of CPV dsRNA replication by destabilizing cypoviral panhandle structure in a similar manner. In accordance with this speculation, we found that when an RNA structure mimicking the cypoviral panhandle was used as a template, HaCPV-5 VP5 effectively promoted primer-dependent transcription initiation by an alternative polymerase (i.e. reverse transcriptase) (Figure 12).

As a heterogeneous group of proteins that share no consensus sequences or motifs, RNA or nucleic acid chaperones are poorly understood in regard to the mechanism(s) governing their ATP-independent helix-destabilizing and annealing stimulation activities. To explain the RNA chaperone activities, an ‘entropy transfer’ model has been proposed. According to this model, RNA chaperones contain intrinsically disordered (unstructured), highly flexible regions that can transfer their disorder or entropy to misfolded RNA molecules on binding to RNA. Such an entropy transfer can destabilize kinetically trapped misfolded RNA molecules, leading to the rearrangement of RNA folding in an ATP-independent manner (13,14). So far, many virus-encoded RNA chaperones, including HIV-1 NC, Vif and Tat, hantavirus N, flavivirus core, coronavirus N and tombusvirus p33, have been predicted to contain intrinsically disordered regions (14,51). However, since the regions responsible for chaperoning activities have not been accurately mapped in many RNA chaperones, the relationship between intrinsic disorder and RNA chaperone activities is difficult to formally determine (43,52). Furthermore, alternative models, such as transient ionic or electrostatic interactions, have also been proposed to explain the RNA chaperone activity (22). Our disorder prediction of CPV VP5 using PONDR VL-XT (51,53,54) indicates that VP5 proteins from three different types of cypoviruses—CPV-5, CPV-1 and CPV-18—contain similar distribution patterns of potentially disordered regions (data not shown), thereby suggesting that the ‘entropy transfer’ applies to HaCPV-5 VP5, and VP5 proteins of other CPVs may also contain RNA chaperone activity.

As a single-shelled member of the family *Reoviridae*, CPV is well recognized as an ideal and simplified model for studying the mechanisms of vRNA replication and mRNA capping of the *Reoviridae* and probably other dsRNA *Viridae*. In this study, we found that a CPV capsid clamp protein VP5 possesses a novel RNA chaperone activity, which is thought to be directly involved in the initiation of CPV dsRNA replication. These findings show that CPV capsid proteins not only include RNA polymerase and mRNA capping enzyme, but also RNA chaperone that is important for vRNA replication and/or translation. This study should extend our understanding of RNA replication of CPV, *Reoviridae* and dsRNA *Viridae*, and also enrich our knowledge about virus-encoded RNA chaperones. Furthermore, since the (+)-vRNA cyclization and panhandle structures commonly exist in the family *Reoviridae*, encoding an RNA chaperone or other RNA remodeling protein, like helicase, might be a common strategy for all reoviruses. Future studies by our group and others should reveal whether rotavirus NSP2, the clamp proteins of other turreted reoviruses, as well as other reoviral structural or nonstructural proteins can also function in 5′-3′ panhandle unwinding and RNA replication initiation.

## SUPPLEMENTARY DATA

Supplementary Data are available at NAR Online.

## FUNDING

This work was supported by National Basic Research Program of China [973 Program, 2014CB542603]; the National Natural Science Foundation of China [31270190 to X.Z., 81201292 to X.Z. and 31270189 to Y.H.]; the Fundamental Research Funds for the Central Universities [204274391 to J.Y.]; the Chinese 111 Project [B06018]. Funding for open access charge: Wuhan University

*Conflict of interest statement*. None declared.

## Supplementary Material

Supplementary Data
