# Supplementary material for: A cypovirus VP5 displays the RNA chaperone-like activity that destabilizes RNA helices and accelerates strand annealing
Source: Nucleic Acids Res. 2013 Dec 5;42(4):2538–54. doi: 10.1093/nar/gkt1256 (PMC3936753; doi:10.1093/nar/gkt1256)
Supplement: Supplementary Data [file supp_42_4_2538__index.html]

A cypovirus VP5 displays the RNA chaperone-like activity that destabilizes RNA helices and accelerates strand annealing — A cypovirus VP5 displays the RNA chaperone-like activity that destabilizes RNA helices and accelerates strand annealing — Supplementary Data 

# A cypovirus VP5 displays the RNA chaperone-like activity that destabilizes RNA helices and accelerates strand annealing

## Supplementary Data

files

**Files in this Data Supplement:**

- Supplementary Data - pdf file
