# Supplementary material for: A cypovirus VP5 displays the RNA chaperone-like activity that destabilizes RNA helices and accelerates strand annealing
Source: Nucleic Acids Res. 2013 Dec 5;42(4):2538–54. doi: 10.1093/nar/gkt1256 (PMC3936753; doi:10.1093/nar/gkt1256)
Supplement: Supplementary Data [file supp_gkt1256_nar-01699-r-2013-File014.pdf]

SUPPLEMENTARY DATA

Supplementary Tables

TABLE S1 PCR primers oligonucleotides used in this study

Primers	Sequence (5'-3')[a]	
VP5-sense	GGATCCCGAGACACAATGGACGATTACTAC	(BamH I)
VP5-anti	AAGCTTGACAGTCTTGAACGACCTAACATGA	(Hind III)
T23A-sense	TCGAATGTCAGCGGCAGAA	
T23A-anti	CTGCCGCTGACATTCGAGATT	
F25A-sense	GCGACAGAAGCTATAAATACAA	
F25A-anti	TATAGCTTCTGTCGCTGACATTCG	
Q273A-sense	GGGCACTGACGTTTGAATTGAT	
Q273A-anti	TTCAAACGTCAGTGCCCAGAAT	

[a] Underlined characters indicate restriction endonuclease sites

TABLE S2 Oligonucleotides used in this study

Name	Sequence (5'-3')[a]
RNA1	5*-GUGUAGUAAUCGUCCAUUGUGUCU-3
RNA2	5-AGACACAAUGGACGAUUACUACAC-3
RNA3	5-AGUUUAAAACGCACGAGACACAAUGGACGAUUAC UACACCAACAGAUCAAUUGA-3
RNA4	5-AGACACAAUGGACGAUUACUACACCAACAGAUCAAUUGA-3
RNA5	5-AGUUUAAAACGCACGAGACACAAUGGACGAUUACUACAC-3
RNA6	5-AGACACAAUGGACGAUUACUACACCAA-3
RNA7	5-AGACACAAUGGACGAUUACUACACCAACAGA-3
RNA8	5-AGACACAAUGGACGAUUACUACACCAACAGAUCA-3
RNA9	5-AGACACAAUGGACGAUUACUACACCAACAGAUCAAUUG AUGGAUUCA-3
RNA-46	5*-GCGGAUAACAAUUUCACACAGGAAACAGCUAUGACCA UGAUUACGA-3
RNA10	5-GUGUAGUAAUCGUCCAUUGUGUCU-3
RNA-146	5-CGGCAAGUGGACGAUUAUCUCCAGAGGAUCGCCGGGAAC CGAGGACGAGUUCGUAAUCAUGGUCAUAGCUGUUUCCUGU GUGAAAUUGUUAUCCGCUCACAAUUCCACACAACAUACGA GCCGGAAGCAUAAAGUGUAAAGCCUGG-3
DNA1	5*-GTGTAGTAATCGTCCATTGTGTCT-3
DNA2	5-AGTTTAAAACGCACGAGACACAATGGACGATTACTACACC AACAGATCAATTGA-3
DNA3	5-AGACACAATGGACGATTACTACAC-3

[a] * indicate HEX labeled site

Supplementary Figures

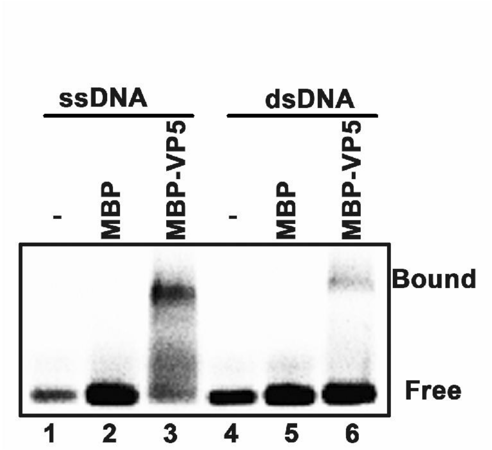

Figure S1. MBP-VP5 has ssDNA and dsDNA binding activity.

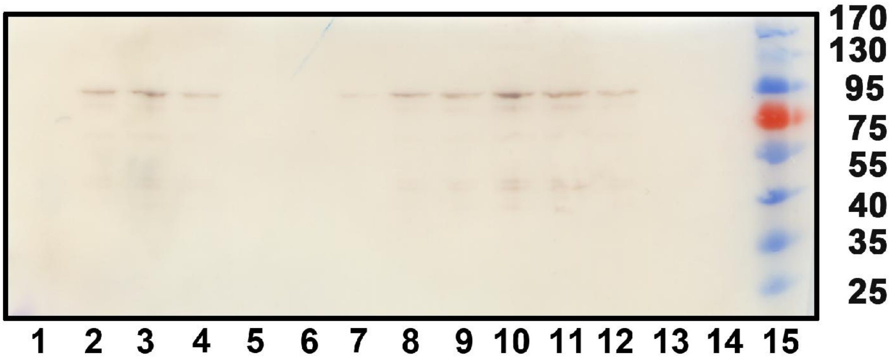

Figure S2. The gel filtration assay of MBP-VP5

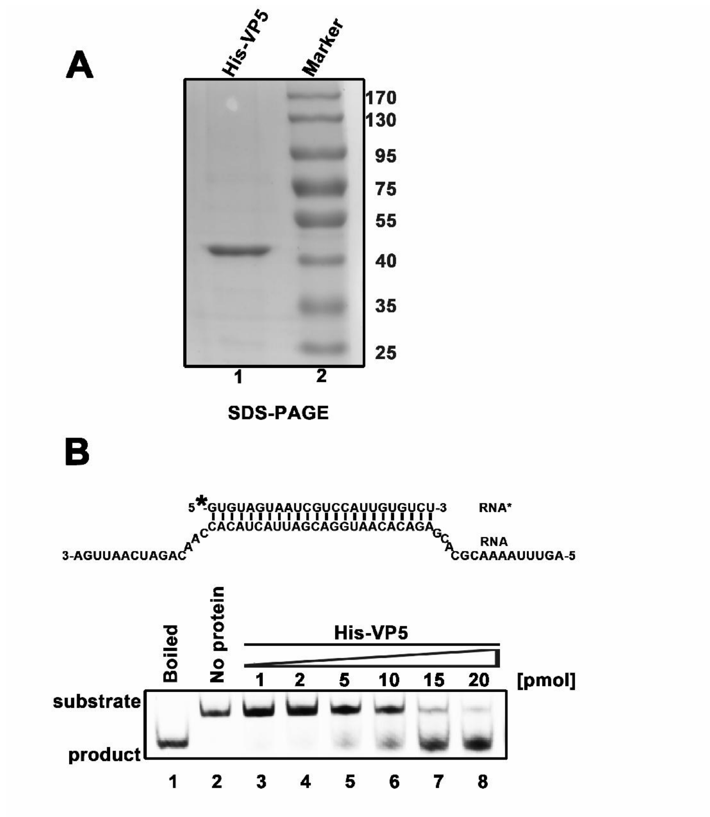

Figure S3. The purification and helix-destabilizing activity of His_6-tagged VP5.

Supplementary Figure Legends

Figure S1. MBP-VP5 has ssDNA and dsDNA binding activity. 10 pmol MBP-VP5 were incubated with 0.1 pmol HEX-labeled ssDNA (DNA1) or dsDNA (DNA1/DNA3) for 30 min. Lanes 1 and 4, no protein supplemented; lane 2 and 5, 10 pmol MBP supplemented; lanes 3 and 6, 10 pmol MBP-VP5 supplemented. Protein-bound and free DNA strands are indicated.

Figure S2. The gel filtration assay of MBP-VP5. MBP-VP5 proteins in solution were separated by gel filtration through a Superdex 200 column. Fractions were concentrated and detected by Western blots with anti-MBP antibody. Lanes 1-14, fractions of 57-58 mL, 59-60 mL, 61-62 mL, 63-64 mL, 65-66 mL, 67-68 mL, 69-70 mL, 71-72 mL, 73-74 mL, 75-76 mL, 77-78 mL, 79-80 mL, 81-82 mL, and 83-84 mL. Lane 15, molecular mass marker.

Figure S3. The purification and helix-destabilizing activity of His_6-tagged VP5. (A) Electrophoresis analysis of purified recombinant His_6-tagged VP5 (His-VP5). Protein sample was loaded onto a 12% SDS-PAGE gel and then visualized via Coomassie blue staining. Lane 1, purified His-VP5; lane 2, protein molecular mass markers. (B) 0.1 pmol helix substrate (RNA1/RNA3; upper panel) was reacted with indicated amounts of His-VP5 (lanes 3-8). Boiled reaction mixture (lane 1) was used as positive control, and the reaction mixture without protein supplementation (lane 2) was used as a negative control.

Supplementary Material and Methods

DNA binding assay

DNA binding assay was performed in 25 mM HEPES-KOH (pH 7.5), 100 mM NaCl, in a volume of 10 μl with indicated amount of protein and 0.1 pmol of ssDNA (DNA1) or blunt-ended dsDNA (DNA1/DNA3). DNA1 is HEX-labeled. Reactions were incubated for 30 min at room temperature. The reactions were terminated by the addition of 2.5 μl 5× sample buffer [20mM Tris–HCl (pH 7.5), 30% glycerol, and 0.1% bromophenol blue]. The DNA-protein complexes were separated by electrophoresis on 2% agarose gels, and gels were scanned by a Typhoon 9200 imager (GE Healthcare).

Gel filtration assay

MBP-VP5 protein was suspended in the buffer containing 100 mM HEPES and 50 mM NaCl at pH 7.4, and separated through a Superdex 200 column (GE Healthcare) with the same buffer. Each fraction contains 2 mL eluted sample. Fractions were concentrated and detected by Western blots with anti-MBP antibody.

Plasmid construction and protein purification

A cDNA fragment of HaCPV-5 RNA segment 8 ORF (VP5) (Accession No. DQ178180) was inserted into the vector pFastBac^{TM}HTB (Invitrogen). The constructed plasmids were subjected to the Bac-to-Bac system (Invitrogen) to express His_6-tagged VP5 (His-VP5) proteins. The recombinant His_6-tagged protein was

purified according to our standard protocol (29).

Supplementary Reference

29. Qi, N., Cai, D., Qiu, Y., Xie, J., Wang, Z., Si, J., Zhang, J., Zhou, X. and Hu, Y. (2011) RNA binding by a novel helical fold of B2 protein from wuhan nodavirus mediates the suppression of RNA interference and promotes B2 dimerization. *J Virol*, 85, 9543-9554.
